# Differential biological responses of adherent and non-adherent (cancer and non-cancerous) cells to variable extremely low frequency magnetic fields

**DOI:** 10.1038/s41598-022-18210-y

**Published:** 2022-08-20

**Authors:** Maryam Sadat Nezamtaheri, Bahram Goliaei, Seyed Peyman Shariatpanahi, Alireza Madjid Ansari

**Affiliations:** 1 Laboratory of Biophysics and Molecular Biology, Departments of Biophysics, Institute of Biochemistry and Biophysics, University of Tehran, 16th Azar St., Enghelab Sq., P.O. Box 13145-1384, Tehran, Iran; 2 Integrative Oncology Department , Breast Cancer Research, Motamed Cancer Institute, ACECR, Vanak Sq., P.O. Box 1517-964311, Tehran, Iran

**Keywords:** Biophysics, Environmental sciences

## Abstract

Extremely low-frequency electromagnetic field (ELF-EMF) induces biological effects on different cells through various signaling pathways. To study the impact of the ELF-EMF on living cells under an optimal physiological condition, we have designed and constructed a novel system that eliminates several limitations of other ELF-EMF systems. Apoptosis and cell number were assessed by flow cytometry and the Trypan Blue dye exclusion method, respectively. In vitro cell survival was evaluated by colony formation assay. The distribution of cells in the cell cycle, intracellular ROS level, and autophagy were analyzed by flow cytometer. Suspended cells differentiation was assessed by phagocytosis of latex particles and NBT reduction assay. Our results showed that response to the exposure to ELF-EMF is specific and depends on the biological state of the cell. For DU145, HUVEC, and K562 cell lines the optimum results were obtained at the frequency of 0.01 Hz, while for MDA-MB-231, the optimum response was obtained at 1 Hz. Long-term exposure to ELF-EMF in adherent cells effectively inhibited proliferation by arresting the cell population at the cell cycle G2/M phase and increased intracellular ROS level, leading to morphological changes and cell death. The K562 cells exposed to the ELF-EMF differentiate via induction of autophagy and decreasing the cell number. Our novel ELF-EMF instrument could change morphological and cell behaviors, including proliferation, differentiation, and cell death.

## Introduction

An enormous increase in electronic device usage has raised a growing concern about the hazardous effects of extremely low-frequency magnetic field (ELF-EMF) on human health. ELF-EMF with frequencies ranging between 0 and 300 Hz is frequently generated by man-made sources including electrical appliances and equipments^1,2^. According to previous studies on childhood leukemia risk, due to residential ELF-EMF exposure^3–5^, ELF-EMF has been classified into group 2B (potentially/carcinogenic to humans)^4,6,7^. At the same time, many studies have reported the positive effects and therapeutic uses of ELF-EMF e.g. in wound repair^8,9^, bone repair, pain management^10^, and Alzheimer’s disease (AD)^11^. Recent in vitro and in vivo studies have shown that exposure to ELF-EMF directly influenced human cells where it induced various effects; for instance, altered reactive oxygen species (ROS) levels^12^, increased intracellular Ca^2+^^13,14^, changed expression of genes involved in cell cycle, metabolism, autophagy, apoptosis pathways^15–17^, and inhibited proliferation^18,19^. Furthermore, ELF-EMF has shown antitumor potency in many types of cancers by inducing increased sensitivity to apoptosis and inhibition of cancer cell proliferation^20^.

Effects of ELF-EMF exposure on different tissues vary according to the field's frequency, amplitude, exposure time^21^ and cell type^22^. However, due to limitations of the field generating equipment, the effects of the physical parameters of ELF-EMF on cell responses has not been thoroughly investigated. Interest in the potential therapeutic effects of ELF-EMF on cancer cells has led to the construction of various devices with different capabilities.

In addition to the frequency and amplitude ranges covered by different ELF-EMF generating systems, the ability of long time exposures in an optimal culture condition is very significant. Therefore, many researchers have tried to use the magnetic field generating equipment in incubators. However, the setups have their own limitations to be used in incubators. For instance, Helmholtz coil has relatively large amount of magnetic field escape which results in the field interaction with the metallic structure of the incubators and inhomogeneity of the field in the exposure volume.

The current study reports a new setup that generates a magnetic field in a wide range of amplitudes (0–150 mT) and frequencies (0–70 Hz). Moreover, considering the significance of studying the biological effects of extremely low-frequency magnetic fields in optimal cell culture conditions, the setup provides a uniform field within the exposure area with negligible field escape which facilitates its ability to be placed within an incubator with the least field interaction with the incubator metallic structure. In fact, the main goal was to introduce a facility for studying the bio-effects of magnetic fields under an optimal physiological condition in living cells. To date, non-ionizing LF and ELF-EMF have been assigned and approved as interventional phenomena in medical, physiological, and biological conditions.

LF weak electromagnetic fields have been known to improve blood oxygenation, circulation cell metabolism, and function. Also, the treatment of pain and fatigue from fibromyalgia^23^, resistant major depressive disorder^24^, and probable reduction of multiple sclerosis symptoms^25^have been reported and proved as neurological effects of such fields. These experiments improve the hypothetical possibility of using ELF-EMF devices for other medical approaches in the near future while there is much evidence that raises such possibilities to be a solution for more severe human disorders. Following our experiments and many other preclinical studies, it has been revealed that the frequency-dependent manner of field biological and physiological effects could be a prospective aspect of using such devices as probable futuristic cancer adjuvant/neoadjuvant therapy devices.

However, more relevancy investigation, frequency-effect characterization assays, and, validity/specificity evaluations are needed to fill the gap between bench to bedside though.

Furthermore, to examine the capabilities of the device, we have studied the bio-effects of magnetic field on the various cell lines with different origins.

Moreover, using the new setup, it is shown how ELF-EMF characteristics influences cellular responses in different cell lines. The results provide new insights into the molecular and cellular mechanisms induced following ELF-EMF exposure in biological systems.

## Materials and methods

### Magnetic field generator facility structure

#### Coil and magnet core

**Figure 1:**
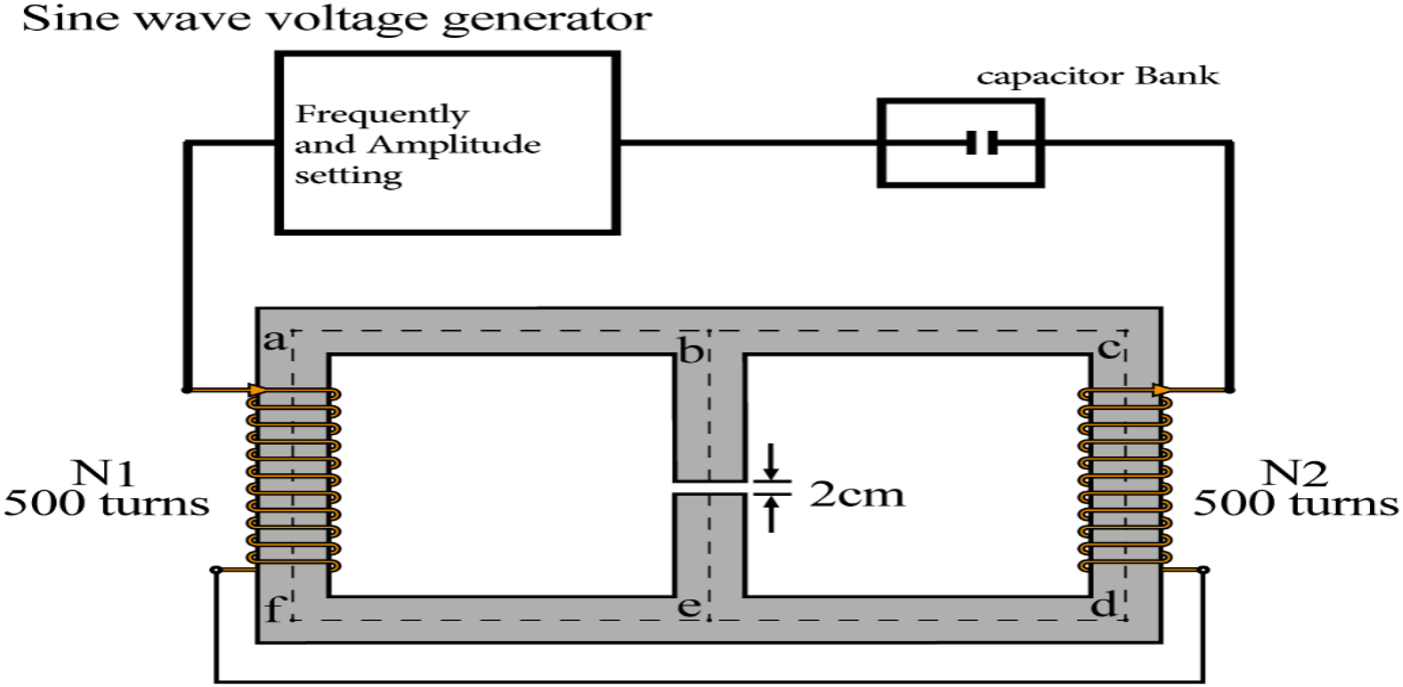
Schematic of the magnetic circuit producing the magnetic field in the air gap.

Figure 1 shows the magnetic circuit used to generate the magnetic field. The magnetic flux produced by the total wiring circuit is conducted to the air gap by the iron core and a maximum amplitude magnetic field is created in the air gap. The small length of the gap (2 cm), relative to the size of the core (square with a 10 cm side), produces a relatively homogenous field. The electrical current of the wiring is generated by an amplified sine wave generator. The frequency and the amplitude of the current can be set on the generator. The magnetic field in the air gap is proportional to the current and is once calibrated using a precise gauss meter.

In order to provide a wide range of frequencies, considering the large 2H inductance of the coil-core system, a capacitor bank is designed to resonate with the inductor in different frequencies in an LC resonator circuit. The bank includes a wide range of capacitors from milli Farad to few Farads. Different combinations of the capacitor could provide different frequencies with no missing gap in the range 0–70 Hz.

**Figure 2:**
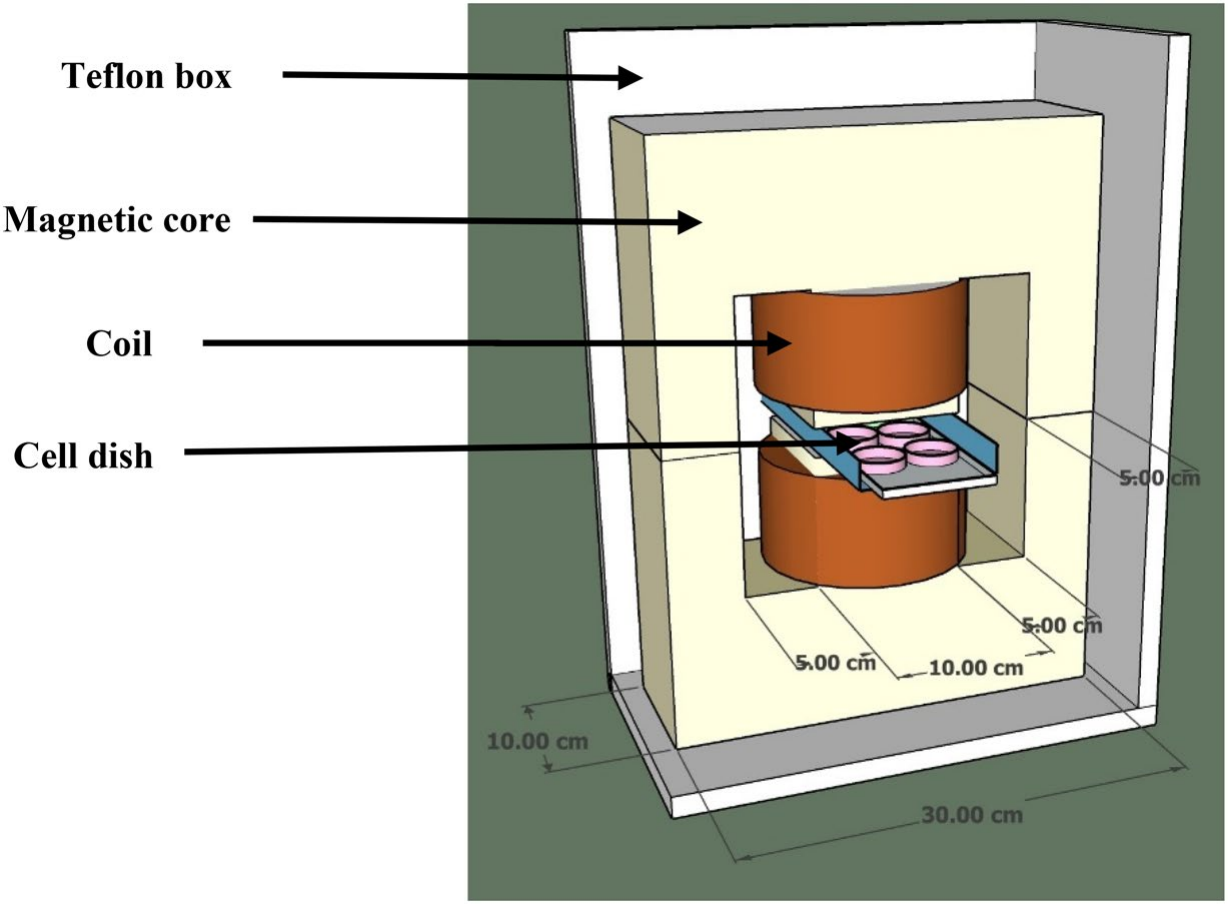
Internal schematic diagram of ELF-EMF exposure system.

Figure 2 shows a schematic diagram of the coil consisting of 2 connected coils in series (with wires of 1.5 mm diameter), with a total number of 1000 turns. The magnetic core consists of laminated cores of stacks of thin sheets of silicon steel coated with an insulating layer. The coil and the magnetic core form a magnetic circuit which produces a uniform magnetic field in the 2 cm gap. The coils and the magnetic coil are covered with Teflon sheets to inhibit iron corrosion in the incubator. The Teflon box is sealed so that humidity diffusion is prevented. It should be noted that the device is not able to apply the magnetic field in parallel to the bottom of the cell culture plate which might be significant for adherent cells like the study in Merla et al.^26^.

**Figure 3:**
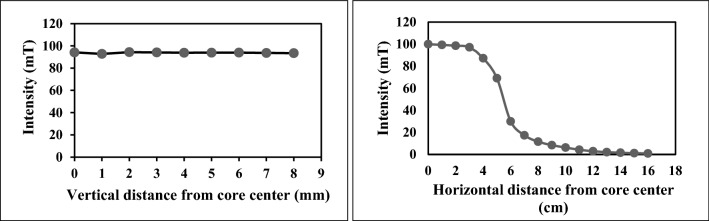
The intensity of the magnetic field at various distances from the core.

Petri dishes at different positions relative to the magnetic core receive various amounts of field intensity. To tackle this issue and exposing all dishes to a uniform magnetic field and to obtain the appropriate location of the Petri dish, we measured magnetic field intensity at different distances from the center of the magnetic core by a Gauss meter (Repco) (Fig. 3).

**Figure 4:**
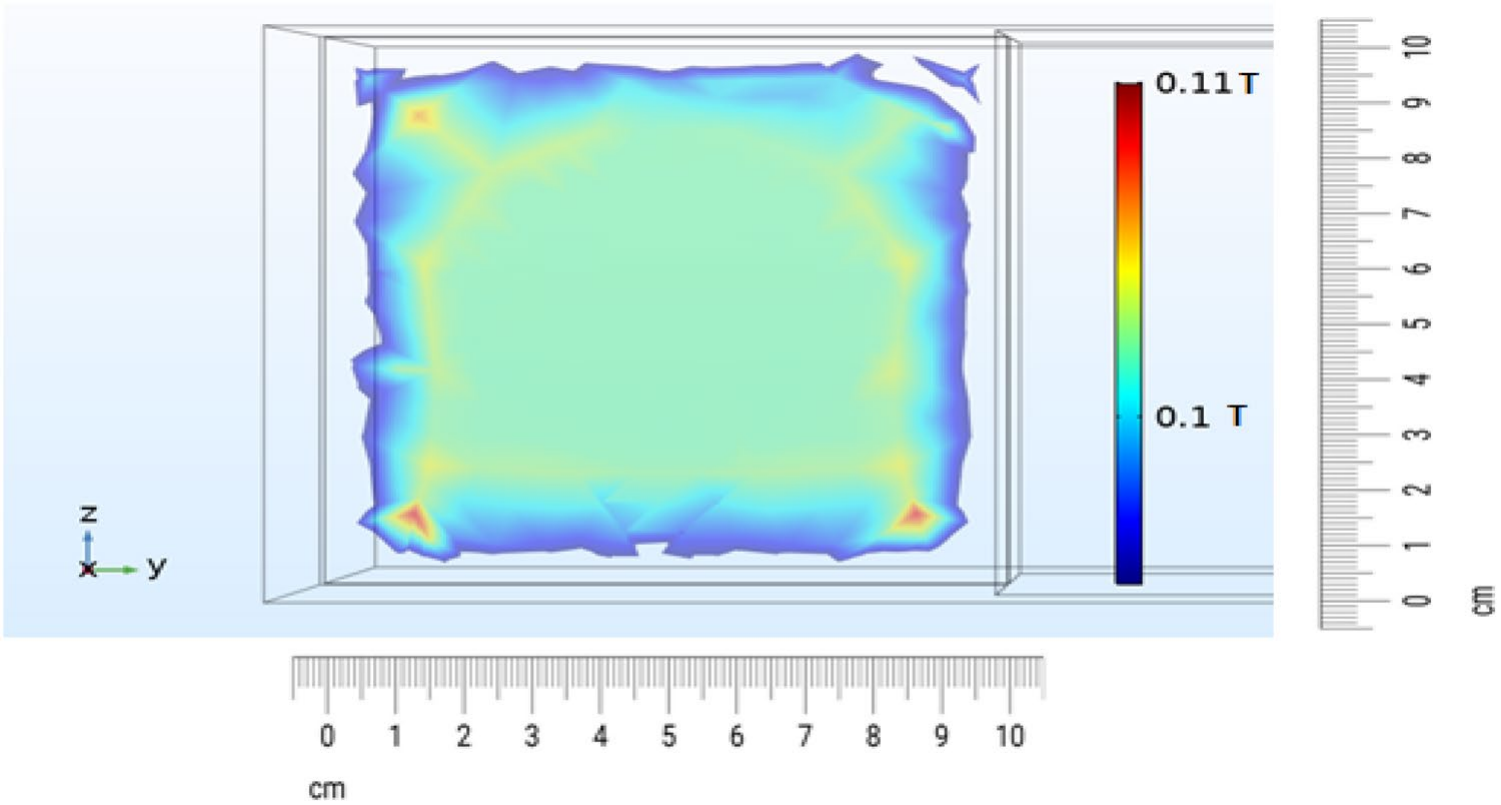
Simulation of the magnetic field intensity within the air gap intersection square with the size of 10 × 10 cm^2^ using COMSOL software.

The measurement showed that the variation of the field intensity within a square of 8 × 8 cm is less than %5. This result is in agreement with the simulation results handled by COMSOL software to find the magnetic field intensity within the gap (Fig. 4).

#### Cooling unit

To remove the generated heat in the core and the coils (resistive and eddy current dissipations), a cold air current is directed into the Teflon box through two silicon hoses, so that the hot atmosphere in the box is removed from the incubator. The cold air current is produced by blowing air current through a radiator cooled with circulating 5 °C circulating water. The temperature in the exposure volume is kept 37 °C for the optimal cell culture condition.

#### ICNIRP guidelines for probable induced electrical field effects

The purpose of the device is to investigate the mechanisms of the magnetic field effects on living cells. ICNIRP guidelines restrictions of low frequencies (1 Hz-3 kHz) induced electric field effects are 0.8 V/m and 0.4 V/m in “All tissues of head and body” for “occupational exposure” and “general public exposure” respectively^27^.

For a 35 mm Petri dish the induced electric field can be calculated theoretically by Faraday’s law^28^. At the device’s maximum magnetic field intensity and frequency values (150 mT and 70 Hz) the induced electric field was about 0.5 V/m. For the whole range of our variable magnetic field exposure system the induced electric field is less than 0.5 V/m (almost less than the restriction values in ICNIRP).

**Figure 5:**
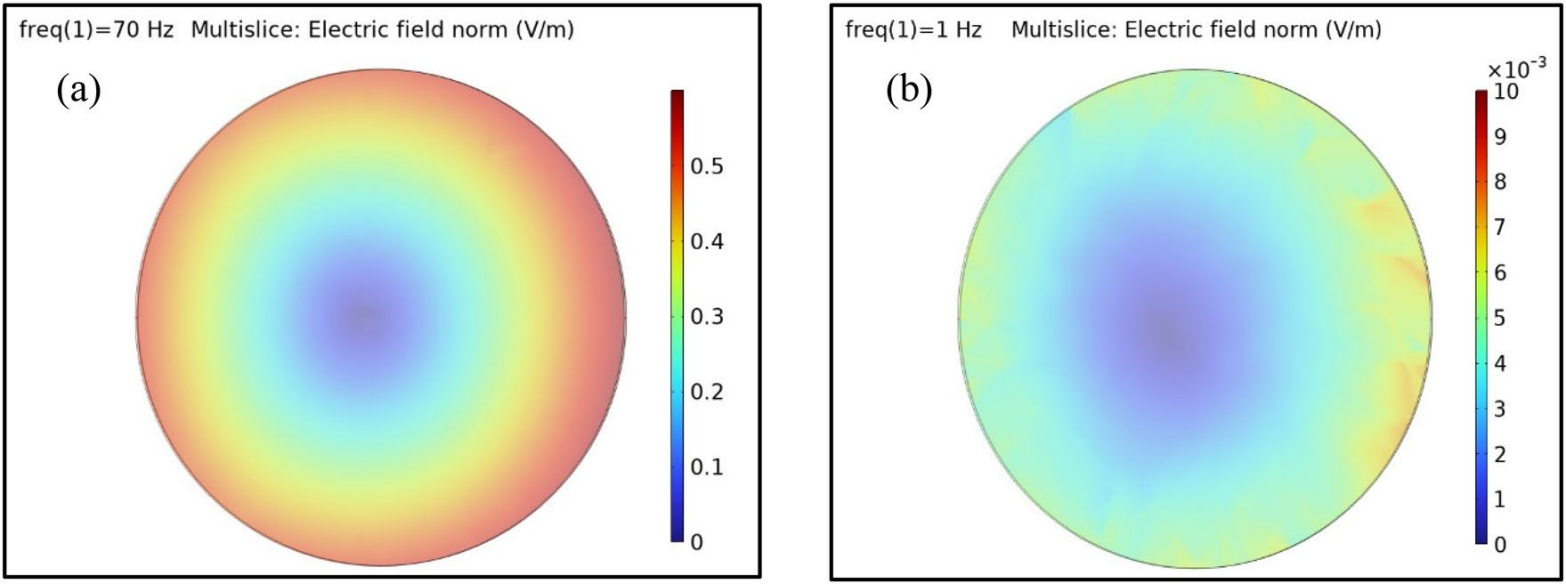
Simulation of the induced electric field for a 35 mm Petri dish using COMSOL software. Plot showing (**a**) the induced electric field for 70 Hz and 150 mT, (**b**) the induced electric field for 1 Hz and 100 mT.

Figure 5a shows the simulated induced electric field for the 150 mT and 70 Hz magnetic field. As observed, the field is maximum at the edges of the plate and minimum at the center. Figure 5b shows the electric field intensity values for 1 Hz and 100 mT that we used in our cellular experiments. In this case the estimated electric field intensity at the edges of the plate was about 7*10^−3^ V/m. The induced field intensity is far smaller than the ICNIRP restriction values.

### Cell culture

Different cell lines were used in our experiments including: two adherent cancer cell lines (Human prostate carcinoma cells (DU145), human breast adenocarcinoma cells (MDA-MB-231)), an adherent non-cancer cell line (Human umbilical vein endothelial cells (HUVEC)) and one non-adherent cancer cell line (Human chronic myelogenous leukemia cells (K562)). Cells were procured from Iranian Biological Resource Center (IBRC, Iran). MDA-MB-231, K562, and DU145 cells were cultured in the RPMI 1640 culture medium (Gibco, United Kingdom) and HUVEC cells cultured in Dulbecco’s Modified Eagle Medium (DMEM) culture medium (Gibco, United Kingdom) supplemented with 10% FBS (Gibco, United Kingdom), penicillin, and streptomycin (1%) (Gibco, USA). Cells were incubated at 37 °C in a humidified atmosphere of 5% CO_2_ (Heraeus, D6450 Hanau) and sub-cultured every 3–4 days.

### Exposure conditions

Various cell lines with different origin were selected to evaluate the dependency of ELF-EMF influence to cell type. For each treatment procedure control cells were also placed in the incubator to receive a magnetic field equal to the background. Therefore, this system provided equivalent environmental parameters for exposed and control cells. Cells were incubated overnight before being exposed to the ELF-EMF. The performance of the new system was examined using different frequencies (0.01 Hz, 0.1 Hz, 1 Hz, 10 Hz) and intensities (1 mT, 10 mT, 100 mT) of the magnetic field at various exposure times (2 h, 24 h, 48 h, 72 h, 120 h) to induce apoptosis-mediated cell death in in-vitro cultures. Then, we evaluated biological responses by selecting the frequency, intensity, and exposure time that yielded the most prominent effect for each cell line. For DU145, HUVEC, and K562 cells, the optimum results were obtained at a frequency of 0.01 Hz, while for MDA-MB-231, the optimum response was acquired at a frequency of 1 Hz. A significant effect was observed only at the intensity of 100 mT and exposure time of 120 h for all groups of cells examined here.

### Cell death assays

#### Apoptosis assay

To perform apoptosis assay, cells were seeded in 35 mm cell culture Petri dishes (Nunc, Denmark), and allowed adherent cells to attach. After 24 h, cells were exposed to ELF-EMF as described above. Then, cells were collected, and for fluorescence labeling and detection of apoptotic and necrotic cells, binding buffer and Annexin V-FITC (IQ products, Netherlands) were added to cells (20 and 5 μl, respectively). Subsequently, 2 μl of propidium iodide (PI) (1 mg/mL) (Sigma-Aldrich, Germany) was added to each sample and analyzed on a BD FACSCalibur flow cytometer (BD Biosciences, San Jose, CA, USA).

#### Clonogenic survival assay

In order to determine in vitro cell survival, clonogenic assay was performed. Cells were seeded in 35 mm Petri dishes and incubated. Log phase cells were exposed to ELF-EMF. Non-exposed and exposed cells were seeded with the density of 500 cells per dish (for MDA-MB-231, 200 cells per dish). Colonies with more than 50 cells were counted using an optical microscope.

#### Autophagy assay

To assess autophagy, we used Acridine orange (AO) (Hopkins & Williams Ltd, United Kingdom) staining for ELF-EMF exposed cells. The cell cultures were exposed to ELF-EMF for 120 h and non-exposed cells incubated with a culture medium containing 2 μg/mL AO for 15 min at room temperature in the dark. Cells including increased acidic vesicular organelles (AVO) (as a marker of autophagy), were measured with Flow cytometry using FloMax software.

#### Cell cycle arrest assay

After 120 h exposure to ELF-EMF, non-exposed and exposed cells were collected and fixed in 70% ethanol (Merck, Darmstadt, Germany) for 1 h at 4 °C. Then cells were washed twice with PBS and incubated with propidium iodide (10 μg/ml) and RNase A (0.1 mg/ml) (Sigma-Aldrich, Germany) for 30 min at 37 °C. The distribution of cells in the cell cycle was analyzed by Flow cytometer using FlowJo software v 10.2.

### Cell counting and viability assay

For each time, 24 h, 48 h, 72 h, and 120 h after exposure, both non-exposed and exposed cells were collected. The viability of cells was assessed using the Trypan Blue dye exclusion method. The fold change was calculated by dividing the cell number in ELF-EMF exposed cells by non-exposed cells per time point.

### Cell Function and differentiation assays

#### Phagocytosis of latex particles

The phagocytic activity of K562 cells was determined with the ability to ingest protein-coated latex particles (marketed commercially for pregnancy test (Ortho gravindex)). Non-exposed and exposed K562 cells, collected at different time points (12, 24, 48, 72, 120 h), were incubated in the medium containing protein-coated latex particles for 1 h at 37 °C. Cells were mounted on cytospin slides, fixed with methanol (Merck, Darmstadt, Germany), and examined using an optical microscope. Differentiated cells were able to phagocytose protein-coated latex particles. The number of cells that had ingested more than 10 particles was counted by using an optical microscope as differentiated cells.

#### Cell differentiation assay

K562 cells which were exposed to ELF-EMF for 24 h and non-exposed cells were harvested and incubated with an equal volume of nitroblue tetrazolium (NBT) (Sigma-Aldrich, USA) solution containing freshly diluted phorbol 12-myristate 13-acetate (PMA) (Sigma-Aldrich, Deisenhofen, Germany) for 40 min at 37 °C. The cells were mounted on cytospin slides, fixed with methanol, and examined using an optical microscope. The differentiated cells were identified by forming a blue formazan precipitate. The formazan crystals were dissolved with dimethylsulphoxide (DMSO) (Merck, Darmstadt, Germany) and the absorbance was read at 560 nm using a microplate reader.

### ROS evaluation

2′, 7′-Dichlorofluorescin diacetate (DCFH-DA) was used as a fluorescent indicator of intracellular ROS formation. After 120 h of exposure to ELF-EMF, DCFH-DA was immediately added to the fresh Hank's buffered salt solution (HBSS) and were incubated for 45 min. Finally, the fluorescence emission of non-exposed and exposed cells was analyzed through the CyFlow Space Flow Cytometer (Partec, Germany).

### Morphological studies and cell size quantification

To record morphological changes (shape and size of the cells), we used an inverted microscope to track the images of K562, DU145, MDA-MB-231, and HUVEC cells that were exposed to ELF-EMF for 120 h. To quantify the cell sizes, we used ImageJ software to calculate the cell surface area. The fold changes were calculated by dividing the cell surface area in ELF-EMF exposed cells by non-exposed cells.

### Statistical analysis

We analyzed and visualized our results using Microsoft Excel and Prism version 9 software (Graph Pad). Statistical analysis for comparisons between groups was performed using Graph Pad Prism. (*P*-value < 0.05) was considered to indicate statistical significance.

## Results

### Induction of apoptosis by ELF-EMF

**Figure 6:**
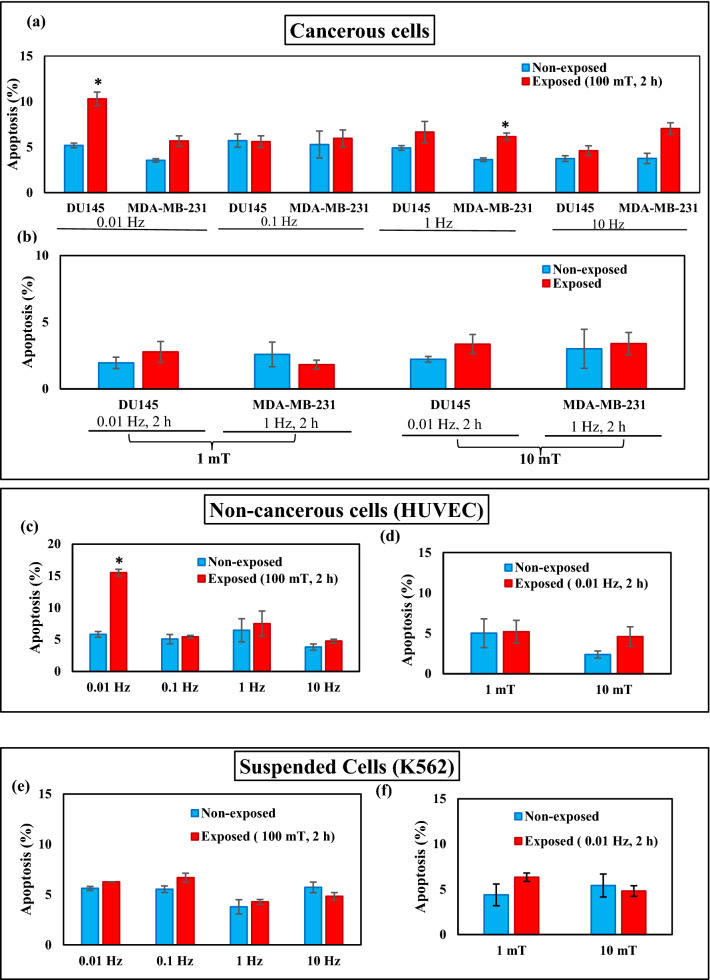

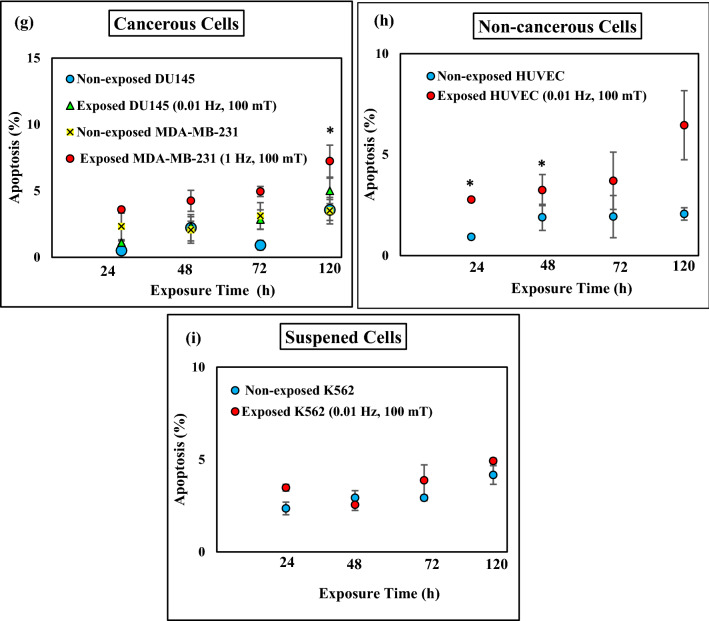
Extremely low frequency electromagnetic field induced apoptosis. ELF-EMF induced apoptosis was examined under different frequencies (0.01 Hz, 0.1 Hz, 1 Hz, 10 Hz) and different intensities (1 mT, 10 mT, 100 mT) and different times (24 h, 48 h, 72 h, 120 h) on HUVEC, DU145, K562 and MDA-MB-231 cell lines. Histograms showing the percentage of apoptotic cells in non-exposed and exposed cells in (**a**) cancerous cells (DU145, MDA-MB-231) with different frequencies (0.01 Hz, 0.1 Hz, 1 Hz, 10 Hz) and intensity 100 mT for 2 h time, (**b**) cancerous cells (DU145, MDA-MB-231) 1 mT and 10 mT for 2 h time. (**c**) non-cancerous cells (HUVEC) with different frequencies (0.01 Hz, 0.1 Hz, 1 Hz, 10 Hz) and intensity 100 mT for 2 h time, (**d**) Non-cancerous cells (HUVEC) with frequency 0.01 Hz and intensity 1 mT and 10 mT for 2 h, (**e**) suspended cells with different frequencies (0.01 Hz, 0.1 Hz, 1 Hz, 10 Hz) and intensity 100 mT for 2 h time, (**f**) suspended cells (K562) with frequency 0.01 Hz, intensity 1 mT and 10 mT for 2 h. Different times (24 h, 48 h, 72 h, 120 h) of exposure in intensity 100 mT for (**g**) cancerous cells (DU145 (0.01 Hz), MDA-MB-231 (1 Hz)), (**h**) non-cancerous cells (HUVEC) frequency 0.01 Hz, (**i**) suspended cells (K562) frequency 0.01 Hz. Bars indicate (Means ± SEM) obtained from three or more independent experiments. Statistical significance between non-exposed and exposed groups was evaluated by a t-test. (*P*-value < 0.05) was considered to indicate statistical significance.

The effects of ELF-EMF highly depend on the nature of the cell line exposed. In this study, we have selected two cancerous cell lines that adhere to the culture plate for growth (DU145 and MDA-MB-231), an adherent non-cancerous human cell line (HUVEC) and a human leukemia cell line that grows in suspension culture media (K562). We performed apoptosis assay under exposure of ELF-EMF with exponentially growing cells. Apoptosis was assessed by the Annexin-V FITC/PI kit and the percentage of dead cells, early apoptotic, late apoptotic, and live cells were determined by flow cytometry. We examined the effects of ELF-EMF frequencies (0.01 Hz, 0.1 Hz, 1 Hz, and 10 Hz) on these cell lines. ELF-EMF- exposed cultures (2 h after exposure, 0.01 Hz and 100 mT) in adherent cancerous cells (DU145) and non-cancerous human cells (HUVEC) showed a significant increase in the fraction of apoptotic cells (the percentages of the early apoptotic cells and the late apoptotic cells combined) by 10% and 15%, respectively (Fig. 6a,c). In MDA-MB-231 cells, another adherent cancerous cell, a moderate rise in apoptosis fraction was observed after 2 h ELF-EMF exposure with 1 Hz and 100 mT intensity (Fig. 6a). Results showed that the ELF-EMF impact on apoptosis induction depended on the field frequency (Supplementary Fig. S1). We selected the frequency that produced the most pronounced effect for each cell line to evaluate other physical parameters. Cells were exposed for 2 h to various intensities of ELF-EMF (1 mT, 10 mT, and 100 mT) with the proper frequency and the fraction of apoptotic cells was determined (Fig. 6b,d,f). A significant effect was observed only at the intensity of 100 mT for all group of cells examined here. Time dependence of apoptosis induction by ELF-EMF exposure was studied by exposing various cell lines under their optimum conditions of frequency and intensity to ELF-EMF for up to maximum of 120 h. Fraction of apoptotic cells increased significantly after 120 h of exposure to ELF-EMF in cancerous cell line (MDA-MB-231) and in non-cancerous cell line (HUVEC) after 24 h and 48 h (Fig. 6g,h). Interestingly, non-adherent cancer cells (K562) did not respond significantly to the ELF-EMF exposure (Fig. 6e,f,i). This result strongly confirm that the effect of ELF-EMF on apoptosis induction depends on cell type.

### Effect of ELF-EMF on cell number

**Figure 7:**
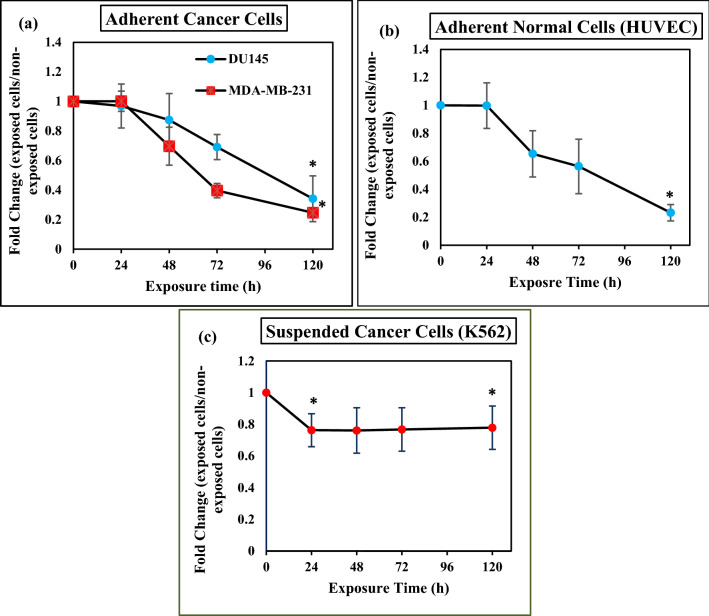
Extremely Low frequency electromagnetic fields decrease cell numbers. Plot showing results of fold change (decrease) following exposure ELF-EMF at long time intensity 100 mT in (**a**) cancerous cells (DU145 (0.01 Hz), MDA-MB-231 (1 Hz)), (**b**) non-cancerous cells (HUVEC) frequency 0.01 Hz in a time-dependent manner. (**c**) plot showing fold change of suspended cells (K562) frequency that was significantly decreased after 24 h exposure and remained constant up 120 h. Decreasing cell number was significant after 120 h exposure in all of the cells. Bars indicate (Means ± SEM) obtained from three or more independent experiments. Statistical significance between non-exposed and exposed groups was evaluated by a t-test. (*P*-value < 0.05) was considered to indicate statistical significance.

The effect of ELF-EMF on cell number was investigated for MDA-MB-231 (1 Hz, 100 mT), DU145, K562, and HUVEC (0.01 Hz, 100 mT) cell lines compared to controls over time with long time exposure. As shown in Fig. 7, this gradual decrease is evident over time and reached the highest level at 120 h with statistical significance difference in cancerous and non- cancerous cell lines (DU145, MDA-MB231, HUVEC). However, non-cancerous HUVEC cells were more sensitive to ELF-EMF after different exposure times at the frequency of 0.01 Hz. Among the different cell lines, only suspended K562 cells showed a statistically significant decrease at 24 h compared to the control K562 cells and remained constant up to 120 h.

### Effect of ELF-EMF on colony formation

**Figure 8:**
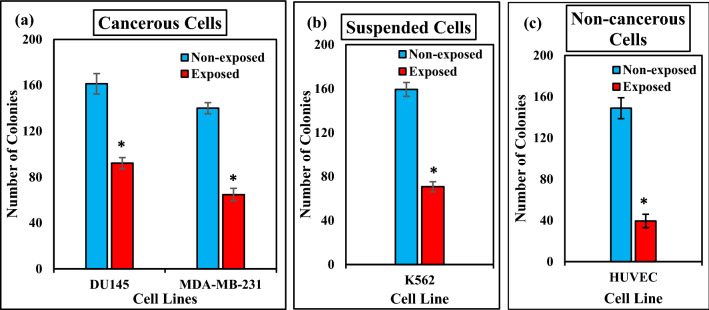
ELF-EMF decreases Clonogenic ability. Plot showing the colony formation of the non-exposed and exposed cells in intensity 100 mT for (**a**) cancerous cells (DU145 (0.01 Hz), MDA-MB-231 (1 Hz)), (**b**) non-cancerous cells (HUVEC) frequency 0.01 Hz, (**c**) suspended cells (K562) frequency 0.01 Hz for 120 h of ELF-EMF exposure. Bars indicate means ± SEM obtained from three or more independent experiments. Statistical significance between non-exposed and exposed groups was evaluated by a t-test. (*P*-value < 0.05) was considered to indicate statistical significance.

To gain further insight into the effects of ELF-EMF on cell growth, we performed colony formation assay after 120 h of ELF-EMF exposure in the MDA-MB-231(1 Hz, 100 mT) DU145, K562, and HUVEC (0.01 Hz, 100 mT) cell lines. Figure 8 shows that ELF-EMF induced a marked reduction in the colony number of all cell lines compared to non-exposed cells (The most significant decrease in non-cancerous HUVEC cells and a minor decrease in adherent cancerous DU145 cells). Together, these findings confirmed that ELF-EMF inhibited the proliferation and colony production of these cell lines.

### Effects of ELF-EMF on the cell cycle distribution

**Figure 9:**
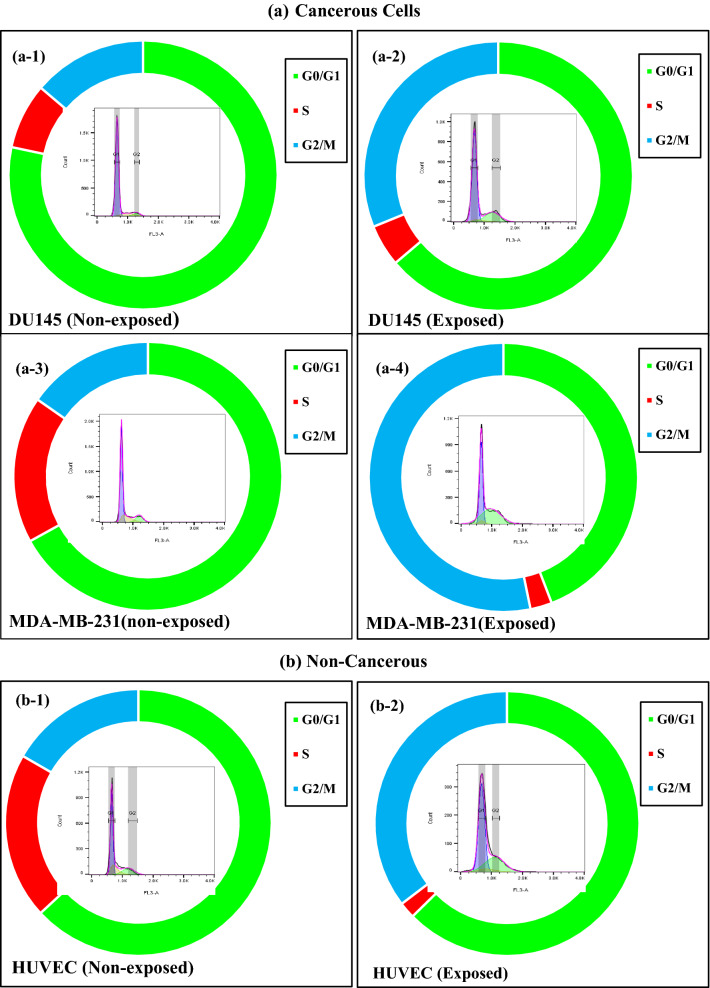

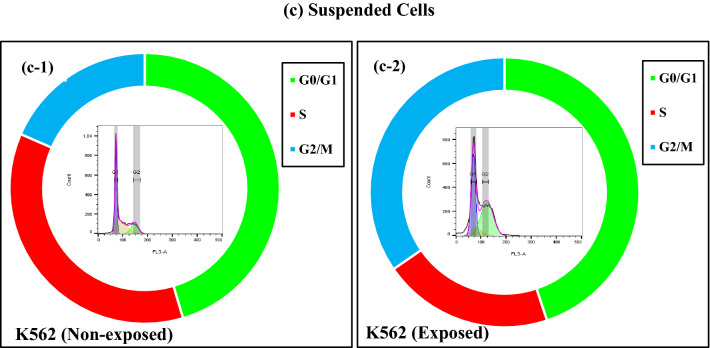
ELF-EMF induced cell cycle arrest. We used Flow cytometry to determine the effect of ELF-EMF on the cell cycle distribution of DU145, K562 and HUVEC cell lines (0.01 Hz, 100 mT, 120 h), MDA-MB-231 cell line (1 Hz, 100 mT, 120 h). Histograms showing the cell cycle distribution of the non-exposed and exposed (**a**) cancerous cells: (a-1) DU145 (Non-exposed, (a-2) DU145 (Exposed), (a-3) MDA-MB-231(non-exposed), (a-4) MDA-MB-231 (Exposed), (**b**) non-cancerous cells: (b-1) HUVEC (Non-exposed), (b-2) HUVEC(Exposed), (**c**) suspended cells: (c-1) K562(Non-exposed), (c-2) K562(Exposed) under ELF-EMF exposure. The histogram in the center shows flow cytometric images of the cell cycle for each cell line.

Cell cycle deregulation at specific stages might lead to aberrant cell proliferation and cancer. To evaluate the relationship between cell proliferation inhibition and cell cycle arrest, we investigated the distribution of cell cycle populations after exposure to ELF-EMF using Flow Cytometry. Our analysis showed ELF-EMF exposure for 120 h in the MDA-MB-231 (1 Hz, 100 mT) DU145, HUVEC, and K562 (0.01 Hz, 100 mT) cell lines led to the accumulation of cells in the G2/M phase (Fig. 9). In the cancerous DU145 cells, we observed a reduced cell number at G0/G1 phases, while in another cancerous cell line (MDA-MB-231 cells), both G0/G1 and S phases were decreased. In non-cancerous HUVEC cells, following exposure to ELF-EMF, cell number at the S phase decreased. Also, in suspended K562 cells, ELF-EMF caused an increase in the number of cells in G2/M accompanied by reduced cell number at the S phase. Our results demonstrated that ELF-EMF decreased the cell number associated with cell cycle arrest, which led to the death of cells.

### Effects of ELF-EMF on K562 cell differentiation

**Figure 10:**
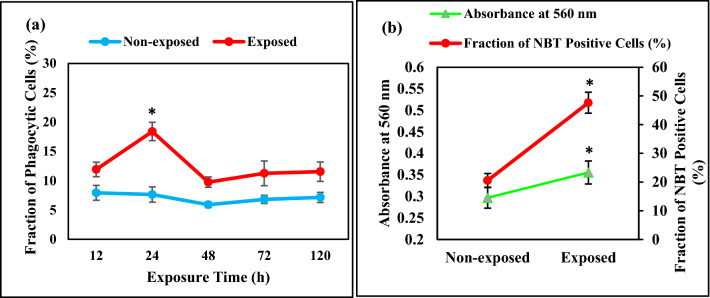
ELF-EMF induced differentiation of K562 cells. Histograms showing: (**a**) The Fraction of K562 cells phagocytizing latex particles in non-exposed and exposed cells of ELF-EMF with frequency 0.01 Hz and intensity 100 mT over time, (**b**) fraction of K562 cells reducing NBT in non-exposed and exposed cells of ELF-EMF (0.01 Hz, 100 mT, 24 h) and absorption of the reduced NBT (formazan) at 560 nm in non-exposed and exposed K562 cells of ELF-EMF (0.01 Hz, 100 mT, 24 h). Bars indicate means ± SEM obtained from three or more independent experiments. Statistical significance between non-exposed and exposed groups was evaluated by a t-test. (*P*-value < 0.05) was considered to indicate statistical significance.

We observed that ELF-EMF (0.01 Hz, 100 mT) decreased cell number in exposed suspended K562 cells after 24 h, colony number was decreased and autophagy was induced after 120 h. Considering the relationship between proliferation inhibition and differentiation induction, ELF-EMF might induce differentiation in suspended K562 cells. To check this hypothesis, we used protein-coated latex particles to examine cell differentiation by phagocytosis properties in multiple exposure times. As shown in Fig. 10a, phagocytosis was mostly observed after 24 h exposure of suspended K562 cells compared to the control. These results suggested that the ELF-EMF was able to induce monocyte/macrophage differentiation in suspended K562 cells. To further validate suspended cells differentiation after exposing cells to ELF-EMF (0.01 Hz, 100 mT, 24 h), we used NBT reduction assay. While undifferentiated K562 cells scarcely reduced NBT, differentiated K562 cells after ELF-EMF exposure reduced NBT and generated blue-black formazan precipitate. Interestingly, formazan deposits were found in about 40% of exposed suspended K562 cells for 24 h (Fig. 10b). We also measured the absorption of the reduced NBT (Formazan) at 560 nm. The mean fluorescence intensity showed a significant increase in exposed K562 cells after 24 h compared to the control K562 cells (Fig. 10b). These results confirmed that ELF-EMF induced monocyte/macrophage differentiation in suspended K562 cells over time.

### Effects of ELF-EMF on the intracellular ROS level

**Figure 11:**
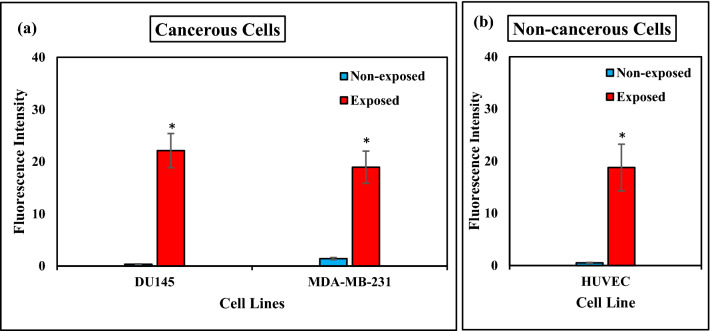
ELF-EMF increases intracellular ROS levels. We used Flow cytometry to determine the effect of ELF-EMF on ROS production by DU145, K562, HUVEC cell lines (0.01 Hz, 100 mT, 120 h), and MDA-MB-231 cell line (1 Hz, 100 mT, 120 h). Bar plot shows the quantitative analysis of the fluorescence intensity in the non-exposed and exposed (**a**) cancerous, (**b**) non-cancerous cells of ELF-EMF stained with DCFH-DA. Bars indicate means ± SEM obtained from three or more independent experiments. Statistical significance between non-exposed and exposed groups was evaluated by the t-test. (*P*-value < 0.05) was considered to indicate statistical significance.

To check if ELF-EMF can change intracellular ROS production, MDA-MB-231(1 Hz, 100 mT) DU145, K562, and HUVEC (0.01 Hz, 100 mT) cell lines, exposed to ELF-EMF for 120 h, were stained with DCFH-DA to assess the ROS level. The intensity of DCF fluorescence in the cancerous and non- cancerous cell lines showed a similar significant increase compared to non-exposed cells (Fig. 11). We did not observe a significant change in intracellular ROS level of suspended K562 cells in response to ELF-EMF exposures.

### Effect of ELF-EMF on autophagic response

**Figure 12:**
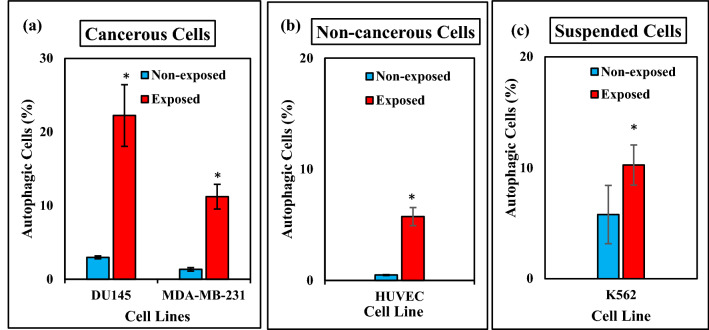
ELF-EMF induced autophagic cell response. We used Flow cytometry to determine the effect of ELF-EMF on autophagy response of DU145, K562, HUVEC cell lines (0.01 Hz, 100 mT, 120 h), and MDA-MB-231 cell line (1 Hz, 100 mT, 120 h). Bar plot showing the quantitative analysis of flow cytometric results non-exposed and exposed (**a**) cancerous, (**b**) non-cancerous, (**c**) suspended cells under ELF-EMF exposure stained with AO fluorescent dye. Bars indicate means ± SEM obtained from three or more independent experiments. Statistical significance between non-exposed and exposed groups was evaluated by a t-test. (*P*-value < 0.05) was considered to indicate statistical significance.

The effect of ELF-EMF on the induction of autophagy in MDA-MB-231 (1 Hz, 100 mT), DU145, K562, and HUVEC (0.01 Hz, 100 mT) cell lines was analyzed using flow cytometry. To evaluate autophagic responses after ELF-EMF exposure for 120 h, we used AO fluorescent dye to stain cells. As depicted in Fig. 12, exposure to ELF-EMF resulted in a significant increase in acidic vesicular organelles and induced autophagic response in the cancerous, non- cancerous and suspended cell lines. Compared to the data obtained from autophagy responses in investigated different cells, a direct relationship between the different autophagic responses and cell types was observed.

### Effect of ELF-EMF on cell morphology

**Figure 13:**
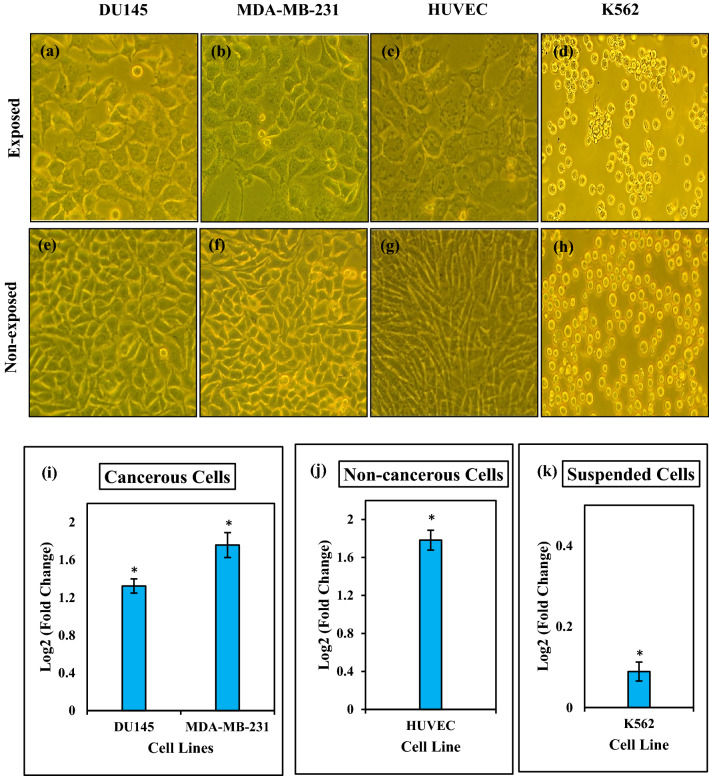
ELF-EMF can stimulate morphological changes. The representative images under an optical microscope (32X) show morphology changes in (**a**) DU145, (**b**) MDA-MB-231, (**c**) HUVEC, (**d**) K562 exposed to ELF-EMF with an intensity of 100 mT at 0.01 Hz for 120 h and MDA-MB-231 exposed to ELF-EMF with an intensity of 100 mT at 1 Hz for 120 h. Images show the morphology of (**e**) non-exposed DU145 cells, (**f**) non-exposed MDA-MB-231 cells, (**g**) non-exposed HUVEC cells, (**h**) non-exposed K562 cells. Histograms show quantified cell size changes following ELF-EMF exposure in (**i**) cancerous, (**j**) non-cancerous and (**k**) suspended cells. Bars indicate means ± SEM obtained from three or more independent experiments. Statistical significance between non-exposed and exposed groups was evaluated by a t-test. (*P*-value < 0.05) was considered to indicate statistical significance.

After 120 h exposure to ELF-EMF, we monitored cells under the microscope and found that the ELF-EMF changed cell morphology in adherent cells (non-cancerous HUVEC cells and cancerous DU145 and MDA-MB-231 cells) and increased the size of cells. We tacked images of the DU145, K562, HUVEC and MDA-MB-231 exposed cell lines to ELF-EMF and quantified cell size by measuring the cell surface area. The images of these cells showed that ELF-EMF induced morphological changes (shape and size of the cells) in the cancerous and non- cancerous cell lines (Fig. 13). Image analysis revealed that the size of all cell lines increased after exposing to ELF-EMF (Fig. 13i,j,k). Among the studied cells, suspended K562 cells showed a minor increased cell size compared to non-exposed cells.

## Discussion

Evaluating the effects of ELF-EMF and assessing the significant role of intensity, frequency, and treatment time on cancer cells has been limited due to the absence of facilities that are capable to adjust important parameters, including physical factors, heterogeneity of exposure, and cell culture conditions. Considering the importance of these factors on biological effects, we have designed and developed new instrumentation that can provide a uniform field within the exposure area and can be placed in a CO_2_ incubator.

The superiority of our instrument could be determined when compared to previous systems, such as Helmholtz coils and /or solenoid. While Helmholtz coils produce fields with limited intensities (less than a few milli Teslas) and the other instruments based on magnets have a very limited range of frequencies, our new design can apply various exposure conditions with a broad range of low frequencies (0–70 Hz/0.001 Hz accuracy) and also the maximum intensity of 150 mT. This range of amplitude and frequency can generate interesting non-ionizing, non-thermal effects^29^ which has recently attracted researchers’ attention for their new biomedical applications^10,30,31^. Moreover, as noted above, the ability of long-time exposures in an optimal culture condition is a prominent feature of our new design.

As a futuristic aspect, we can develop this device to have a bigger exposure site in order to conduct in vivo and human investigations as well. The technology of producing vast various spectrums with lower limitations for intensity would be a great opportunity to assess more mechanistic evaluations of ELF-EMF. The results showed that ELF-EMF has the ability to induce apoptosis and decrease cell number at various time points. Also, according to our findings ELF-EMF changed morphology of cells and increased the size of cells after 120 h exposure. The most significant increase was found in non-cancerous HUVEC cells and cancerous MDA-MB-231 cells. However, Makinistian et al. tested the effect of ELF-EMF on U251 and MDA-MB-231 cell lines. They showed that ELF-EMF only inhibited the U251 cell line proliferation without changing morphological features^32^. In another study, Kapri-Pardes in MDA-MB-231 under ELF-EMF at 50 Hz frequency and 1 mT intensity observed no effect on cell proliferation, cell morphology, and cell death^33^.

**Figure 14:**
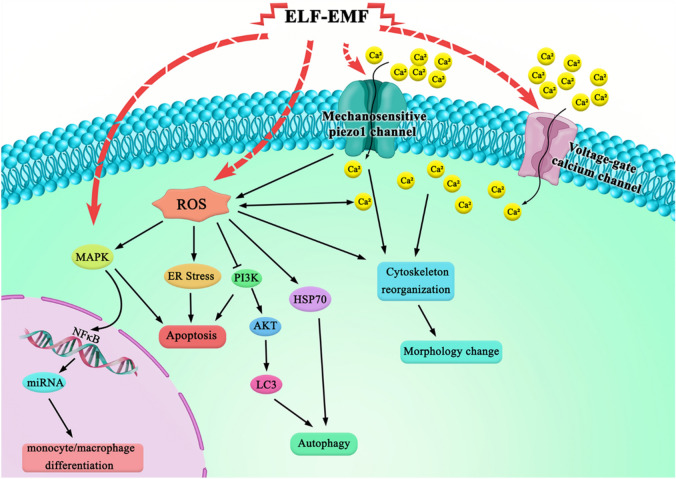
Effect of ELF-EMF on the cell responses. Diagram showing the possible signaling pathways activated by ELF-EMF.

Meanwhile, Hasanzadeh et al. showed that exposure to ELF-EMF (50 Hz, 2 mT, 3 h) altered the morphology of the SH-SY5Y human neuroblastoma cell line and decreased cell proliferation that might be associated with altered gene expression^34^. It is well known that ELF-EMF, as a stress stimulus, can induce morphological changes in cells under stress conditions mainly due to the changes in cell membrane structure and cytoskeletal organization^31,35^. Moreover, ion transports at the cell membrane and osmotic stress can fine-tune the cell volume^36,37^. Besides, mechanical forces between cells and extracellular matrix (ECM) can change cell morphology, function, spreading, and volume^38,39^. There are several investigations in which the effect of ELF-EMF on voltage gated calcium channels has been reported that would lead to increase intracellular calcium levels^40,41^. Although, there is no evidence of the ELF-EMF effects on mechanosensitive calcium channels like Piezo1, it could be a possible mechanism to stimulate oxidative stress and morphological alterations, cell swelling through cytoskeleton re-organization, as well. It has been shown that the function of the mechanosensitive channels may also depend on ECM and osmotic stress^42^. Additionally, changes in cell morphology and cytoskeleton organization might be associated with increased intracellular ROS and calcium levels^43,44^. Therefore, we suggest that activating mechanosensitive channels under ELF-EMF and subsequent increased ROS and calcium level might link our observed ELF-EMF-induced morphological changes to stress-related cell responses and cytoskeleton reorganizations (Fig. 14). There has been a developing body of evidence revealing that ELF-EMF has various effects on cellular processes and functions, including important signaling pathways e.g., apoptosis, autophagy, proliferation, differentiation, and cell cycle^45^. However, it is not clear how ELF-EMF affects cell response. Apoptosis is essential to maintain the homeostatic balance, and any failure in this process might increase the risk of tumor development^46^. Excessive cell growth and abrogated function of cell cycle factors are the main characteristics of cancer cells. Activation of the cell death signaling pathways and inhibiting cell cycle progression and proliferation are the most effective therapeutic strategies in cancer treatment^47^.

Reported pieces of evidence showing contradictorily observations of proliferating effects of ELF-EMF and apoptotic footprints as well. While a convincing part of the evidence suggests stimulatory impacts after exposure to ELF-EMF^48–52^, some studies reported the inhibitory effects^53,54^. This study provides a novel approach to test the effects of short and long times of ELF-EMF exposure on various cells and a variety of different physical parameters. To achieve this, investigated cells were exposed to ELF-EMF with different extremely low frequencies, intensities, and times for assessing the percentage of apoptotic cells. The numbers of apoptotic cells increased significantly after short and long time exposures to ELF-EMF. Induction of apoptosis by ELF-EMF over different exposure time intervals depends on the evaluated cell line type and the frequency. It has been showed that the cell type specificity has important role in induction of apoptosis. While loss of integrin in cell detachment through modulation of p53 results in DNA damage-induced apoptosis, thus suspended cells are more resistant to such apoptosis^55^.

Furthermore, our results suggest that ELF-EMF decreases cell number in a time-dependent manner in the adherent cell lines. These observations are in line with the previous findings suggesting an inhibitory effect of ELF-EMF on cell proliferation instead of inducing apoptosis^56^.

On the other hand, cell cycle population analysis by flow cytometry showed that ELF-EMF induced a cell-cycle delay by an arrest in the G2/M phase after 120 h in all investigated cell lines. In melanoma B16-F10 cell line, LF-MF (low frequency magnetic field) (0.4 T, 7.5 Hz, 2 h/ day 5 days) inhibited the growth and metastasis and arrested cell cycle by an increase of cell population in the G2/M-phase^52^. The arrest of HUVEC and MS-1 cells in the G2/M phase has been shown to be induced by ELF-EMF of 2 mT intensity and frequency of 50 Hz^57^. ELF-EMF caused cell cycle arrest in the G2/M phase probably by down-regulating the B 1cyclin and CDK1 expressions that act as an accelerator of mitosis phase transition. It has been shown, that in the brain, ELF-EMF exposure of 30 min/day for a period of 10 days can affect free radical production, and an extending exposure time to 60/min/day, caused adaptation to the field^58^. Our results are in line with these discoveries, suggesting that cell cycle arrest without inducing apoptosis can be due to the gradual adaptation of cell culture. This is caused by the prolonged exposure of these cells to stress conditions.

Autophagy is a homeostatic and adaptive process that acts in stress conditions by removing cytoplasmic constituents and regulating many diseases consisting of cancer, infection, and neurodegeneration^59^. Among four different cell types, studied here, all the cells displayed different autophagy responses. These differences could be related to the distinct effect of cell type on the variable expression of stress-related genes. Accumulating evidence shows that ELF-EMF can induce autophagy and show a potent anti-tumor effect^45^. Xu et al.^60^ showed that ELF-EMF induced autophagy causes cell death and suppresses lung cancer through up-regulating miR-486 expression and inhibit AKT/mTOR signaling.

The observed variations in biological responses of ELF-EMF exposure might be due to the distinct effect of cell type on expressing varying stress-related genes. The diagram (Fig. 14) showed that how the ELF-EMF might activate downstream signaling pathways and cellular responses (e.g., apoptosis, autophagy^61^, morphology change, differentiation) through oxidative damage.

Our observations are in agreement with the previous findings suggesting that the influence of ELF-EMF on biological systems might be modulated at particular combinations of frequency, amplitude, and time exposure, phenomena called “window effect”^21,62–64^. The molecular mechanism of the window effect is poorly understood. However, some studies have proposed the increased free radicals generated through the radical pair mechanism (RPM) as a possible explanation^65,66^. Interestingly, we found that exposure of continuous ELF-EMF for 120 h significantly increased ROS levels in adherent cell lines.

A growing body of reports indicates that the cell response to magnetic field exposure varies as a function of frequency and intensity^21^. Importantly, physical parameters of ELF-EMF, including frequency and intensity and time of exposure, could affect cell behavior and cause various biological effects.

Patruno et al. suggested that ELF-MF exposure (50 Hz, 1 mT for 1 h) induced proliferation in human HaCaT keratinocyte by increasing the mTOR pathway (PI3K/Akt) and activation of ERK signaling pathways^67^, while another study by Huang et al. showed that ELF-MF exposure in the same cell line (60 Hz,1.5 mT for 144 h) inhibited cell growth and activated the ATM-Chk2-p21 pathway, resulting in cell cycle arrest at the G1 phase^68^. Kim et al.^69^ showed that repetitive exposure to ELF MF (60 Hz 6 mT for 30 min every 24 h for 3 days) induced DNA double-strand breaks (DSBs) and apoptosis mediated by p38 activation in IMR90 (human lung fibroblast) primary cells and HeLa (human cervical carcinoma) cells. The same group in another study demonstrated that exposure to ELF-MF (60 Hz, 7 mT for 10–60 min) induced DNA DSBs without apoptosis and subsequently activated the DNA damage checkpoint pathway in both non-cancerous and cancerous cells (IMR90 primary cells and HeLa cells) without inducing intracellular ROS production^70^. Benassi et al. found that exposure of Human Neuroblastoma Cells (SH-SY5Y) to ELF-MF (50 Hz, 1 mT for 24/48/72 h) significantly increased ROS level^71^. On the other hand, exposure of SH-SY5Y cells to pulsed electromagnetic field (intensity: 2 ± 0.2 mT; frequency: 75 ± 2 Hz for 10 min, 4 times/week) decreased H_2_O_2_-induced ROS^72^. These controversies are related to a window effect too that has been introduced in literature such as Zhou et al., in which they showed frequency-dependent proliferation responses. Exposure of 6B 1 hybridoma cells to ELF-MF (30 Hz, 0.8 mT for 1 h) significantly inhibited proliferation; although at either lower or higher frequencies, this restriction was highly variable: decreased to zero or even changed to positive values^73^.

Interestingly, Grinland et al. showed the window effect (intensity-dependent) on the kinetics of cell cycle progression. Normal human fibroblasts exposed to ELF-MF (60 Hz, 20 and 200 μT for various times up to 30 h) showed a significant increase in the length of the G1 phase but no significant effect was observed at higher flux densities 2 and 20 mT^21^. These results accompanied by many others indicate that comprehensive studies to investigate wide range effects of different parameters of magnetic field-cells interaction are necessary^11,74^.

Moreover, Srdjenovic et al.^75^ and Khavari et al.^76^ revealed ELF-EMF exposure in K562 cells (50 Hz, 100 μT, 24 h and 48 h) and DU145 cells (50 Hz, 0.6 mT, 24 h and 48 h) significantly increased cell proliferation. However, Yoshizawa et al. showed that exposure of K562 cells to ELF-EMF at 500, 100, 20, and 2 µT intensity and 50 Hz, 60 Hz frequency during 24 h and 48 h does not affect cell proliferation^77^. Our current study showed that ELF-EMF decreased cell number in DU145 cells for a long time and K562 cells significantly reduced cell number at 24 h compared to the control K562 cells.

In the current study, cell differentiation as a critical cell response following ELF-EMF exposure was assessed. Loss of differentiation and uninterrupted division is one of the peculiar features of cancer cells. Therefore, strategies to induce differentiation can be used to postpone cancer cells and eliminate tumor phenotypes. Several experimental studies indicated that exposure to ELF-EMF affects the differentiation of murine and human cells^78^. Ayşe et al.^79^ showed that ELF-EMF (50 Hz, 5 mT, daily 1 h/ four days) along with hemin induction (K562 cells induced by hemin to erythroid differentiation) caused a 20% increase in the differentiated K562 cells while single exposure to ELF-EMF for 1 h decreased cells differentiation. Another study confirmed that the ELF-EMF (2 mT, 50 Hz, 96 h) exposure promotes all-trans retinoic acid (ATRA) -induced acute promyelocytic leukemia NB4 cells to differentiate. This could be due to the ROS production and extracellular signal-regulated kinases (ERK1/2) phosphorylation which could be associated with its change and decreases cellular proliferation potential^80^. In our study, ELF-EMF alone and in the absence of any differentiation promoting factor, induced differentiation of suspended K562 cells to monocyte and/ macrophage through the inhibition of cell proliferation. Phorbol myristate acetate (PMA) has been shown to induce monocytic and megakaryocytic differentiation of K562 cells through the activation of mitogen-activated protein kinase (MAPK) pathway^81,82^. We showed that ELF-MF induced monocytic and/macrophage differentiation of K562 cells. According to the previous studies showing ELF-MF might activate the MAPK cascades^33^, we suggest that MAPK pathway might be involved in K562 differentiation after exposure to ELF-MF (Fig. 14).

## Conclusion

In summary, our current study unveiled the role of ELF-EMF in cellular processes, including change in cell number, differentiation, and cell apoptosis. The effects of the new ELF-EMF instrument on various cells depend on cell types and physical parameters. In this study, we assessed reduction in cell number and inhibition of cell cycle progression and subsequent induction of autophagy and apoptosis. Exposure to ELF-EMF increased intracellular ROS levels, molecules that are involved in many biological processes. Our work showed that ELF-EMF could affect cell morphology and increase the size of cells. The results of suspended K562 cells differentiation indicated ELF-EMF could serve as a potential instrument to cell differentiation. Further investigation is needed to define which molecular mechanisms and signaling pathways are behind of function ELF-EMF that affects cell response.

## Supplementary Information

Supplementary Information.

## Data Availability

The database analyzed during the present study are available from the corresponding authors on reasonable request.
