# Supplementary material for: Differential biological responses of adherent and non-adherent (cancer and non-cancerous) cells to variable extremely low frequency magnetic fields
Source: Sci Rep. 2022 Aug 20;12:14225. doi: 10.1038/s41598-022-18210-y (PMC9392794; doi:10.1038/s41598-022-18210-y)
Supplement: Supplementary file 1 — Supplementary Information. [file 41598_2022_18210_MOESM1_ESM.pptx]

## Slide 1
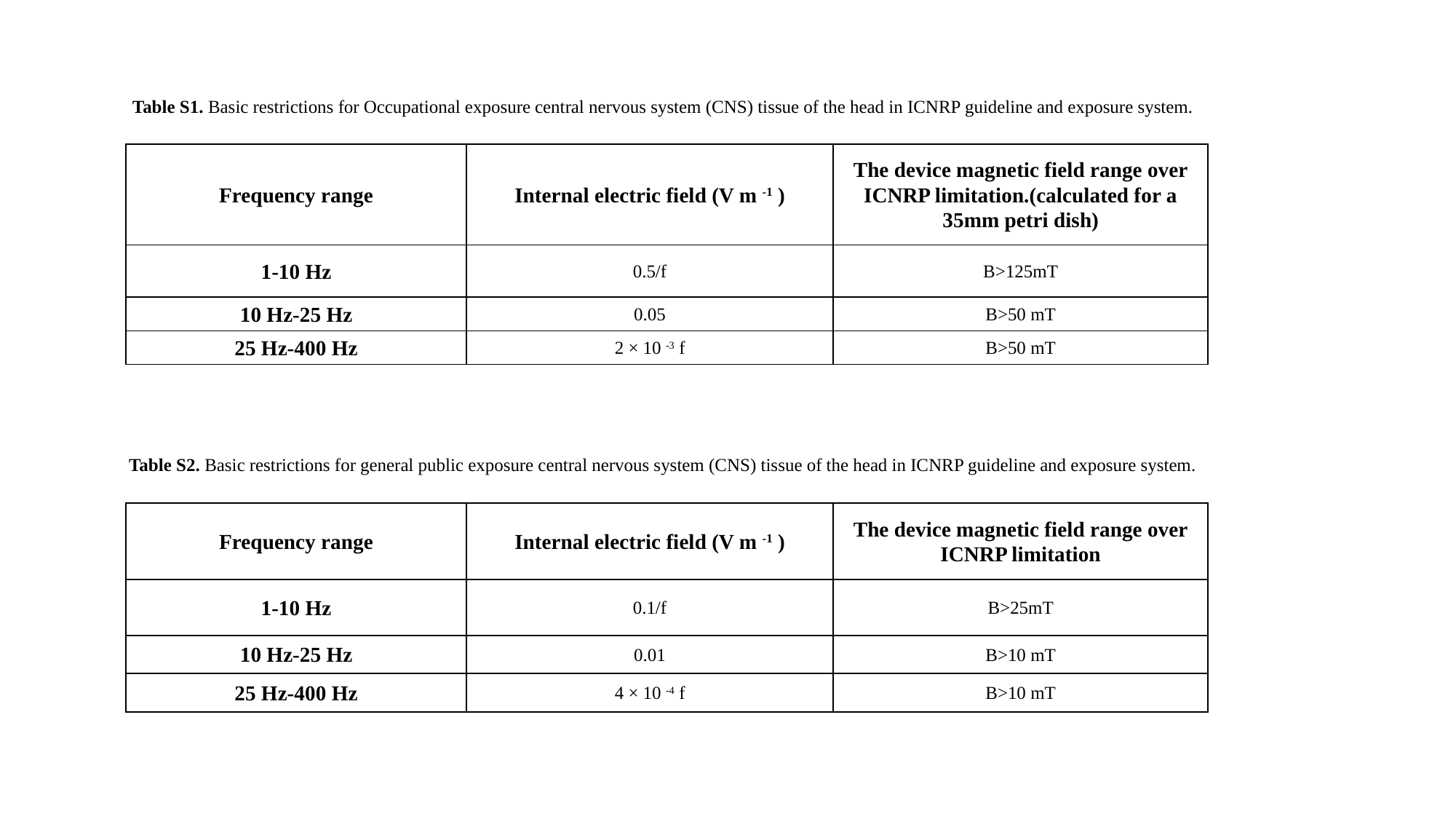

Table S1. Basic restrictions for Occupational exposure central nervous system (CNS) tissue of the head in ICNRP guideline and exposure system.
| Frequency range | Internal electric field (V m -1 ) | The device magnetic field range over ICNRP limitation.(calculated for a 35mm petri dish) |
| --- | --- | --- |
| 1-10 Hz | 0.5/f | B>125mT |
| 10 Hz-25 Hz | 0.05 | B>50 mT |
| 25 Hz-400 Hz | 2 × 10 -3 f | B>50 mT |
Table S2. Basic restrictions for general public exposure central nervous system (CNS) tissue of the head in ICNRP guideline and exposure system.
| Frequency range | Internal electric field (V m -1 ) | The device magnetic field range over ICNRP limitation |
| --- | --- | --- |
| 1-10 Hz | 0.1/f | B>25mT |
| 10 Hz-25 Hz | 0.01 | B>10 mT |
| 25 Hz-400 Hz | 4 × 10 -4 f | B>10 mT |

## Slide 2
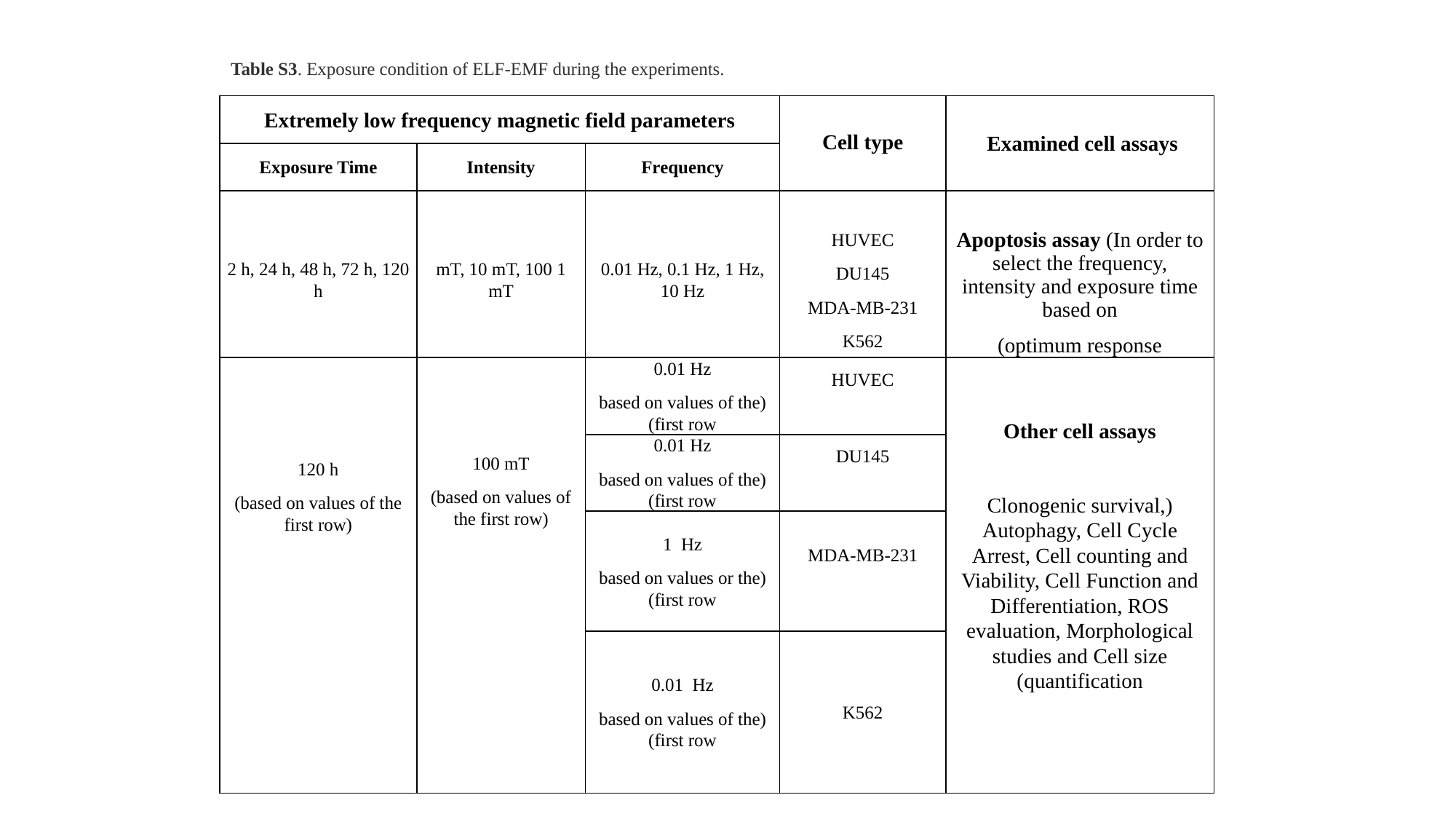

Table S3. Exposure condition of ELF-EMF during the experiments.
| Extremely low frequency magnetic field parameters | | | Cell type | Examined cell assays |
| --- | --- | --- | --- | --- |
| Exposure Time | Intensity | Frequency | | |
| 2 h, 24 h, 48 h, 72 h, 120 h | 1 mT, 10 mT, 100 mT | 0.01 Hz, 0.1 Hz, 1 Hz, 10 Hz | HUVEC DU145 MDA-MB-231 K562 | Apoptosis assay (In order to select the frequency, intensity and exposure time based on optimum response) |
| 120 h (based on values of the first row) | 100 mT (based on values of the first row) | 0.01 Hz (based on values of the first row) | HUVEC | Other cell assays (Clonogenic survival, Autophagy, Cell Cycle Arrest, Cell counting and Viability, Cell Function and Differentiation, ROS evaluation, Morphological studies and Cell size quantification) |
| | | 0.01 Hz (based on values of the first row) | DU145 | |
| | | 1 Hz (based on values or the first row) | MDA-MB-231 | |
| | | 0.01 Hz (based on values of the first row) | K562 | |

## Slide 3
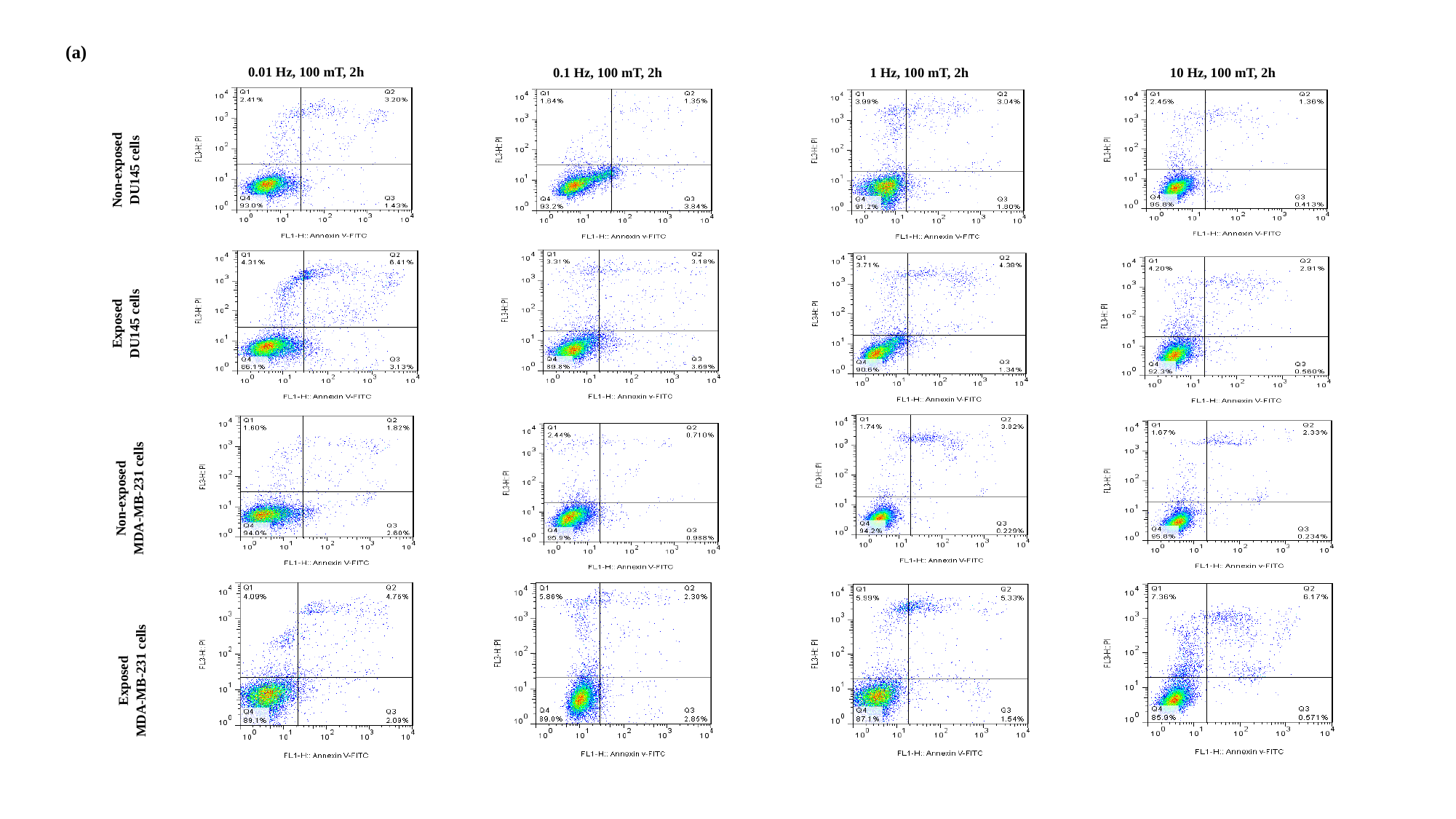

(a)
0.01 Hz, 100 mT, 2h
10 Hz, 100 mT, 2h
0.1 Hz, 100 mT, 2h
1 Hz, 100 mT, 2h
| | | | |
| --- | --- | --- | --- |
| | | | |
| | | | |
| | | | |
Non-exposed
DU145 cells
Exposed
DU145 cells
Non-exposed
MDA-MB-231 cells
Exposed
MDA-MB-231 cells

## Slide 4
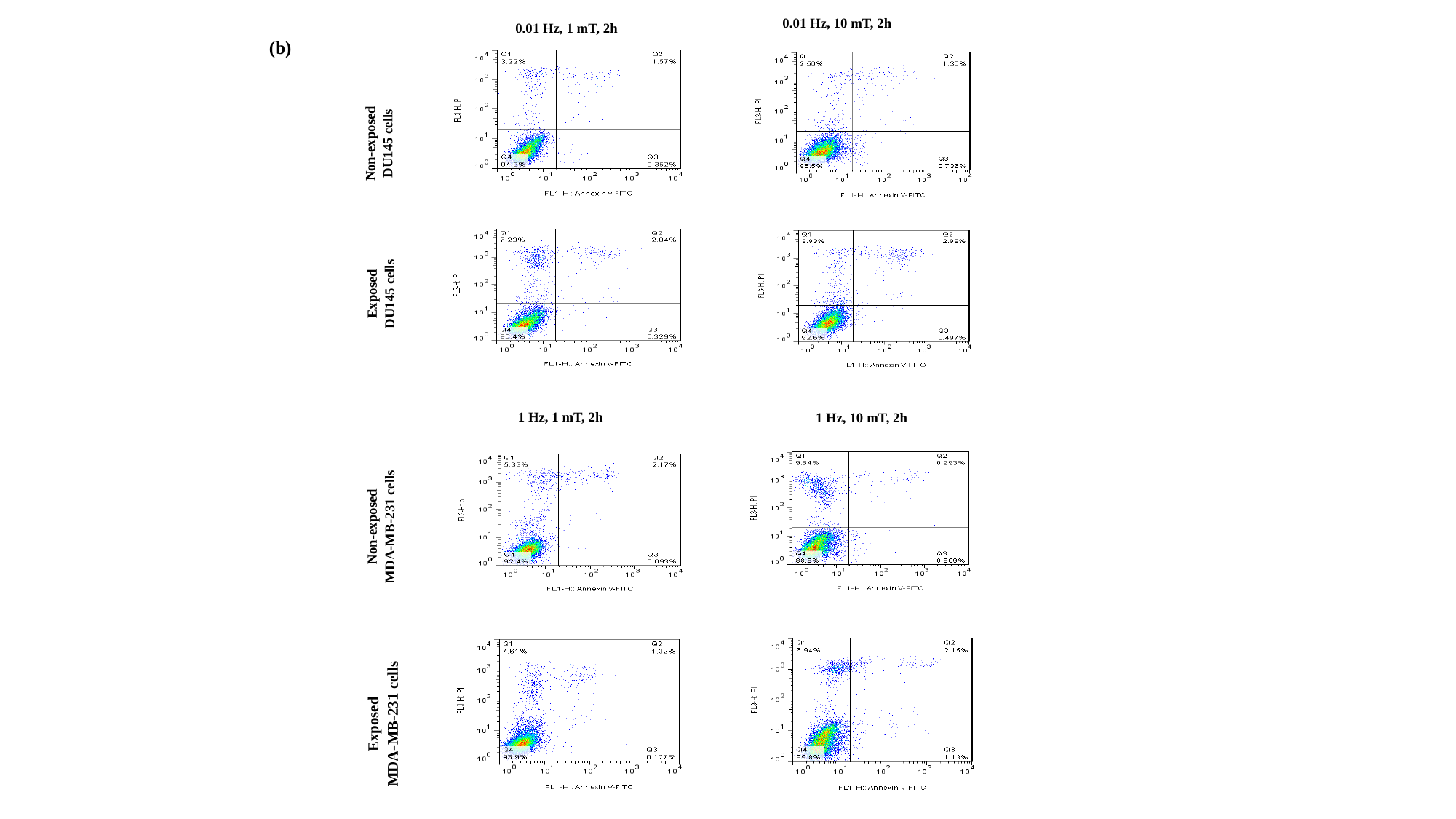

0.01 Hz, 10 mT, 2h
0.01 Hz, 1 mT, 2h
(b)
| | |
| --- | --- |
| | |
Non-exposed
DU145 cells
Exposed
DU145 cells
1 Hz, 1 mT, 2h
1 Hz, 10 mT, 2h
| | |
| --- | --- |
| | |
Non-exposed
MDA-MB-231 cells
Exposed
MDA-MB-231 cells

## Slide 5
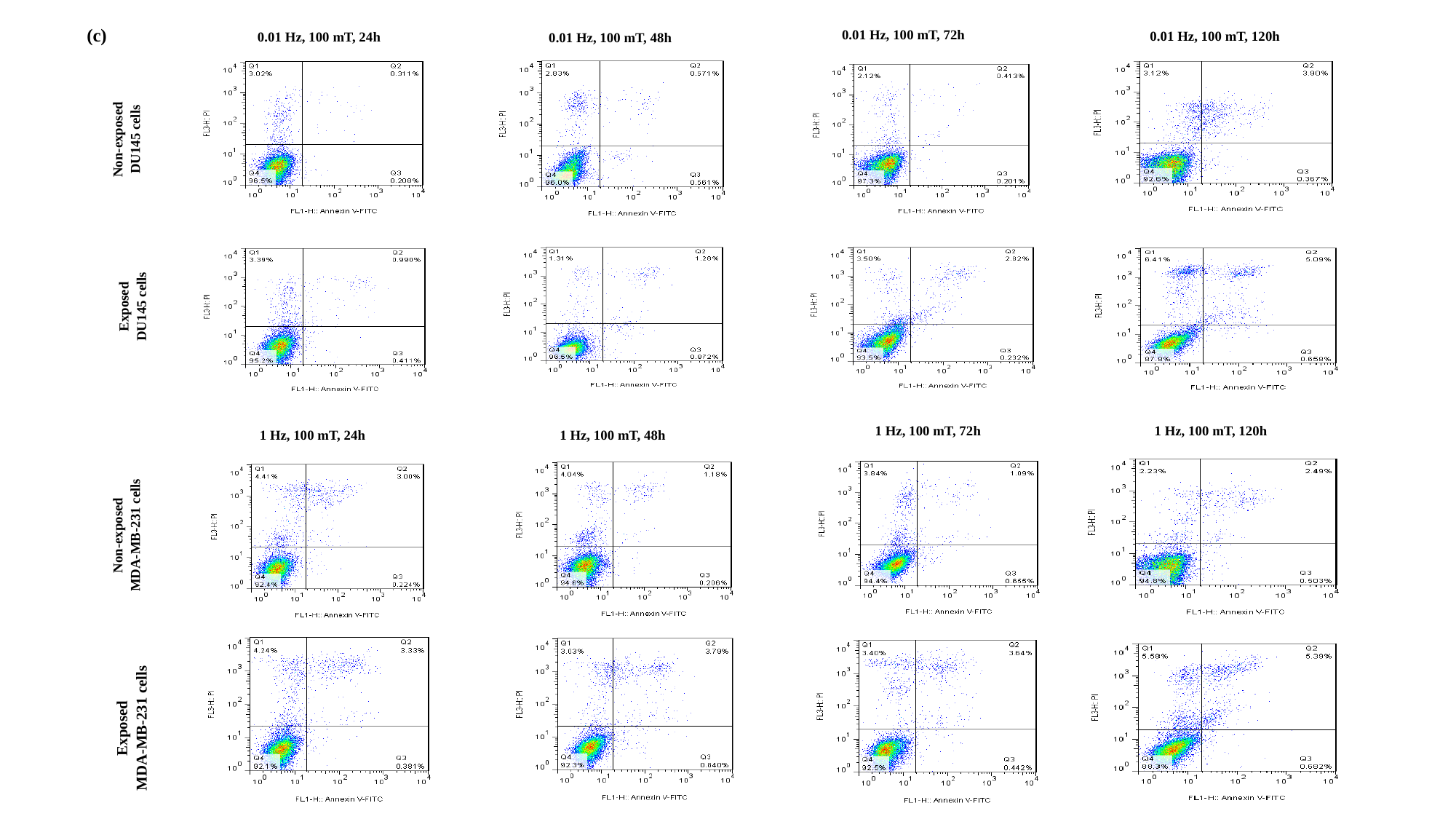

(c)
0.01 Hz, 100 mT, 72h
0.01 Hz, 100 mT, 120h
0.01 Hz, 100 mT, 24h
0.01 Hz, 100 mT, 48h
| | | | |
| --- | --- | --- | --- |
| | | | |
Non-exposed
DU145 cells
Exposed
DU145 cells
1 Hz, 100 mT, 72h
1 Hz, 100 mT, 120h
1 Hz, 100 mT, 24h
1 Hz, 100 mT, 48h
| | | | |
| --- | --- | --- | --- |
| | | | |
Non-exposed
MDA-MB-231 cells
Exposed
MDA-MB-231 cells

## Slide 6
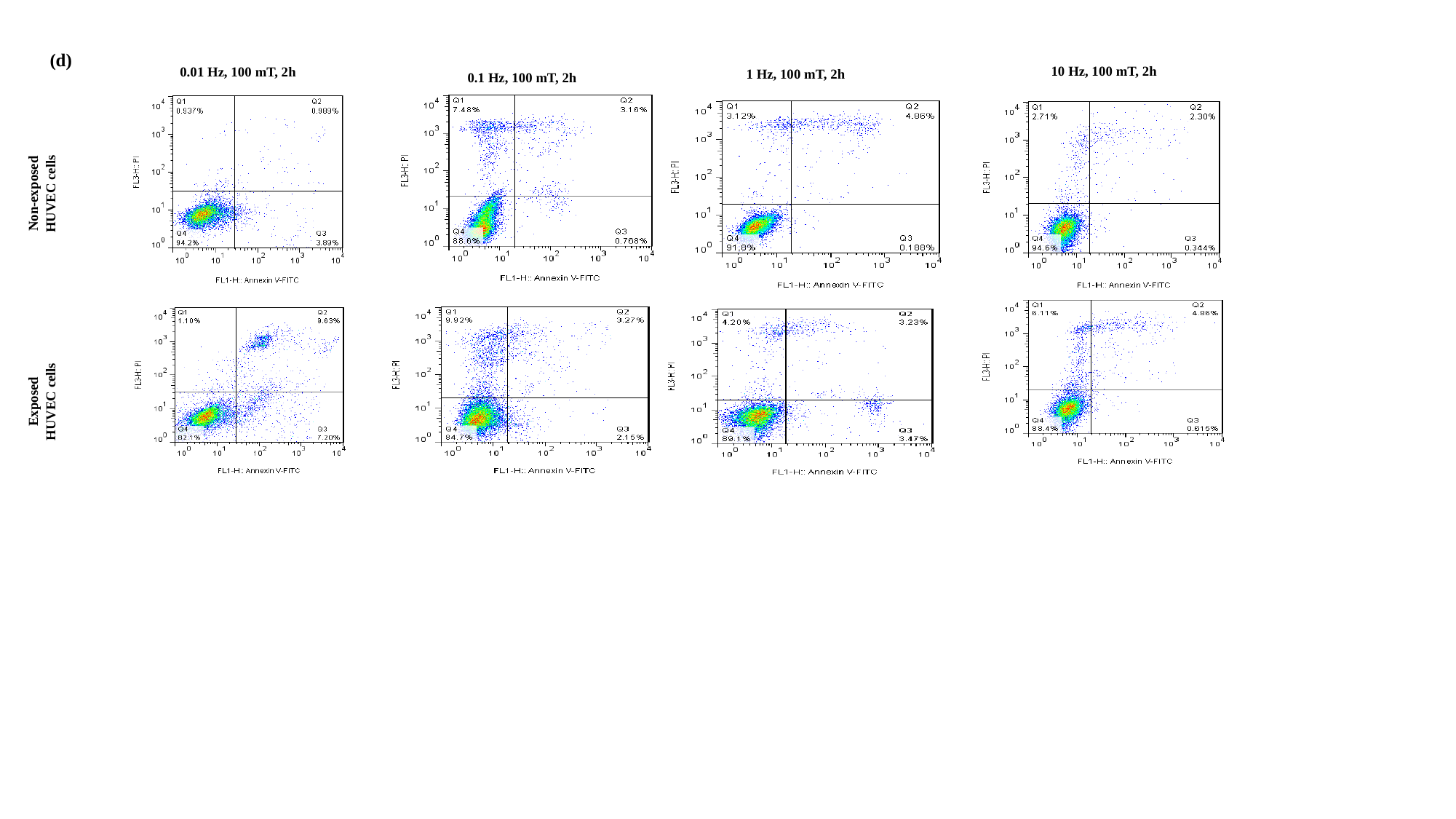

(d)
10 Hz, 100 mT, 2h
0.01 Hz, 100 mT, 2h
1 Hz, 100 mT, 2h
0.1 Hz, 100 mT, 2h
| | |
| --- | --- |
| | |
| | |
| --- | --- |
| | |
Non-exposed
HUVEC cells
Exposed
HUVEC cells

## Slide 7
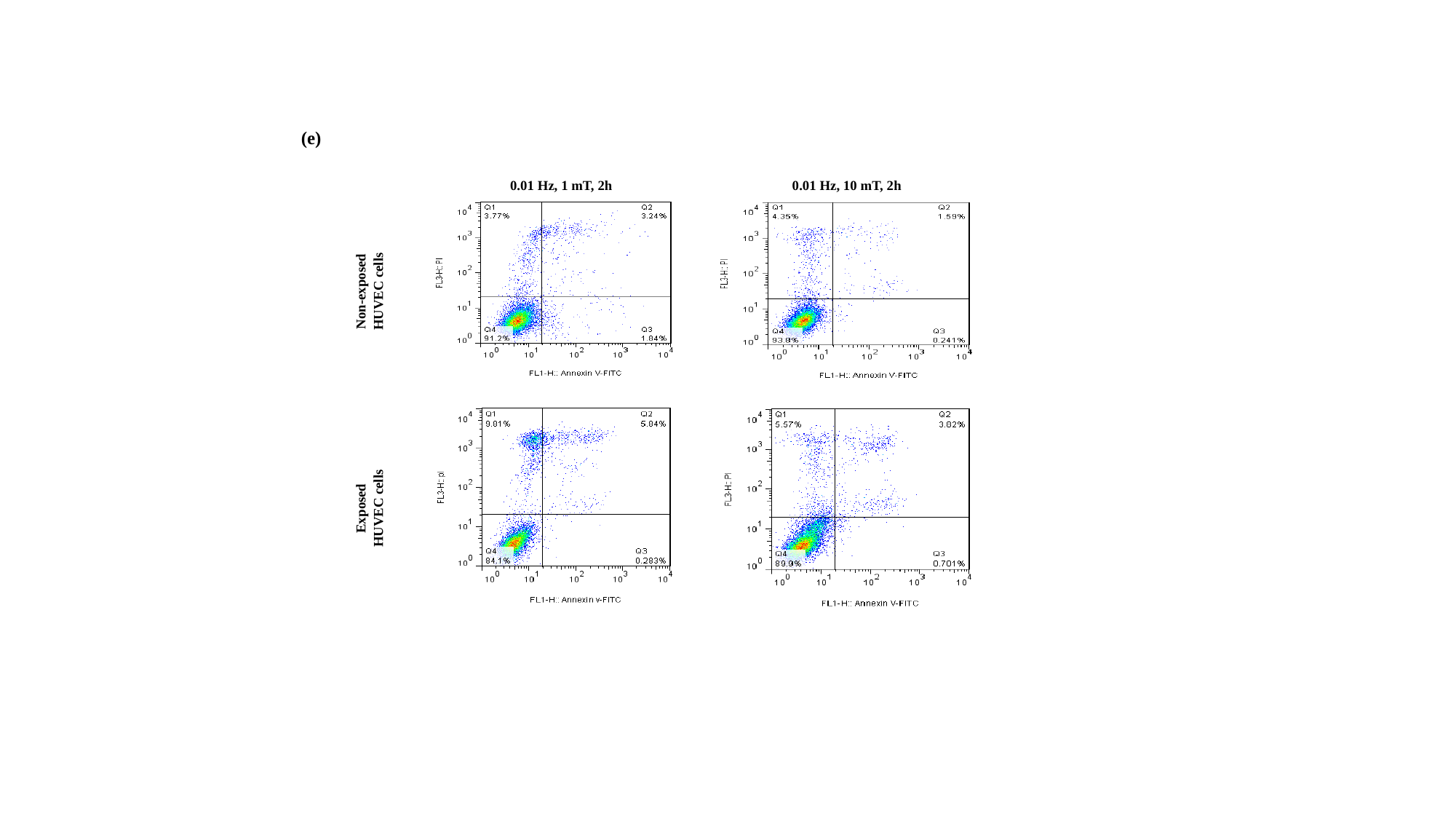

(e)
0.01 Hz, 1 mT, 2h
0.01 Hz, 10 mT, 2h
| | |
| --- | --- |
| | |
Non-exposed
HUVEC cells
Exposed
HUVEC cells

## Slide 8
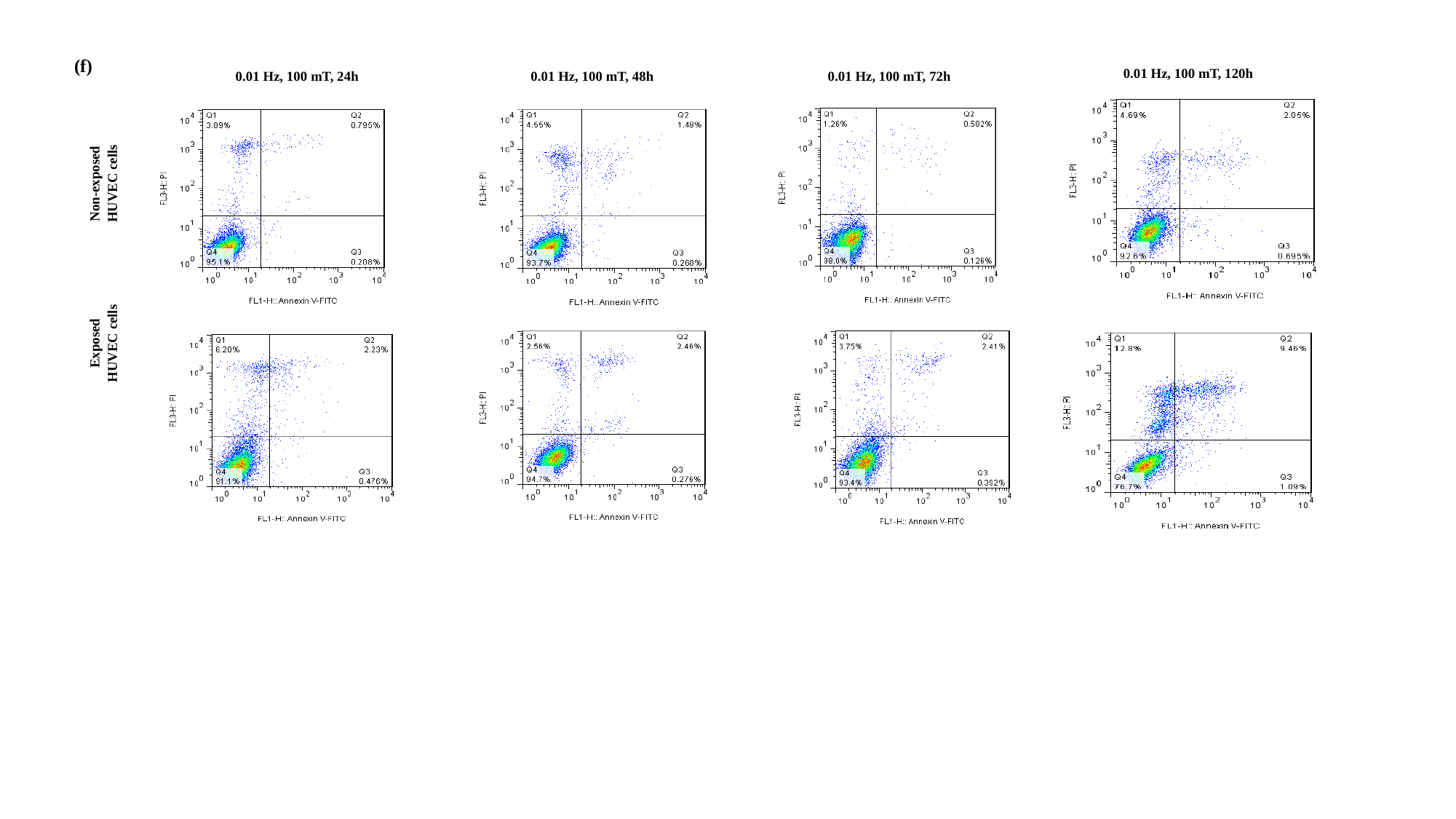

(f)
0.01 Hz, 100 mT, 120h
0.01 Hz, 100 mT, 24h
0.01 Hz, 100 mT, 48h
0.01 Hz, 100 mT, 72h
| | | | |
| --- | --- | --- | --- |
| | | | |
Non-exposed
HUVEC cells
Exposed
HUVEC cells

## Slide 9
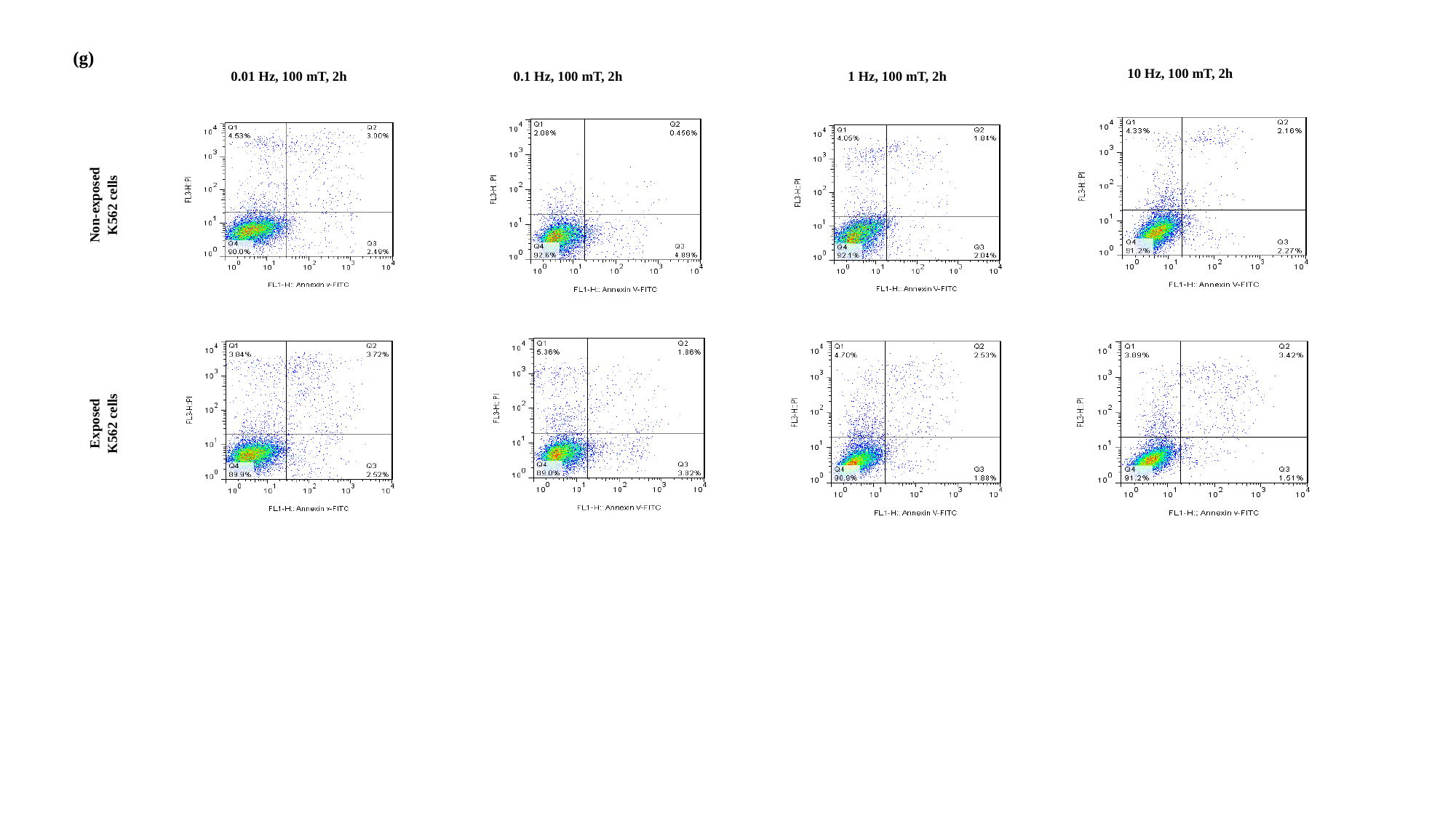

(g)
10 Hz, 100 mT, 2h
1 Hz, 100 mT, 2h
0.01 Hz, 100 mT, 2h
0.1 Hz, 100 mT, 2h
| | | | |
| --- | --- | --- | --- |
| | | | |
Non-exposed
K562 cells
Exposed
K562 cells

## Slide 10
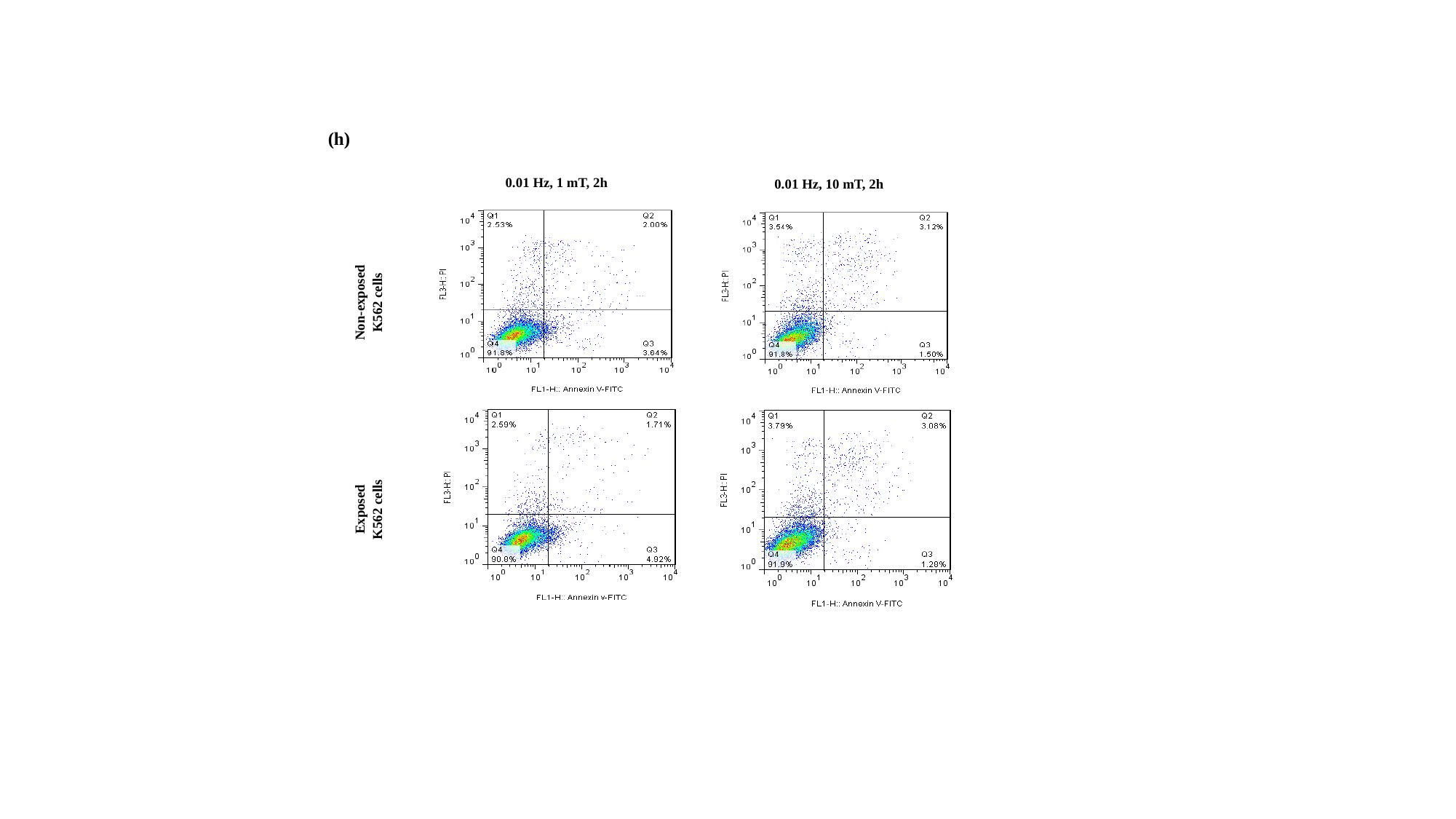

(h)
0.01 Hz, 1 mT, 2h
0.01 Hz, 10 mT, 2h
| | |
| --- | --- |
| | |
Non-exposed
K562 cells
Exposed
K562 cells

## Slide 11
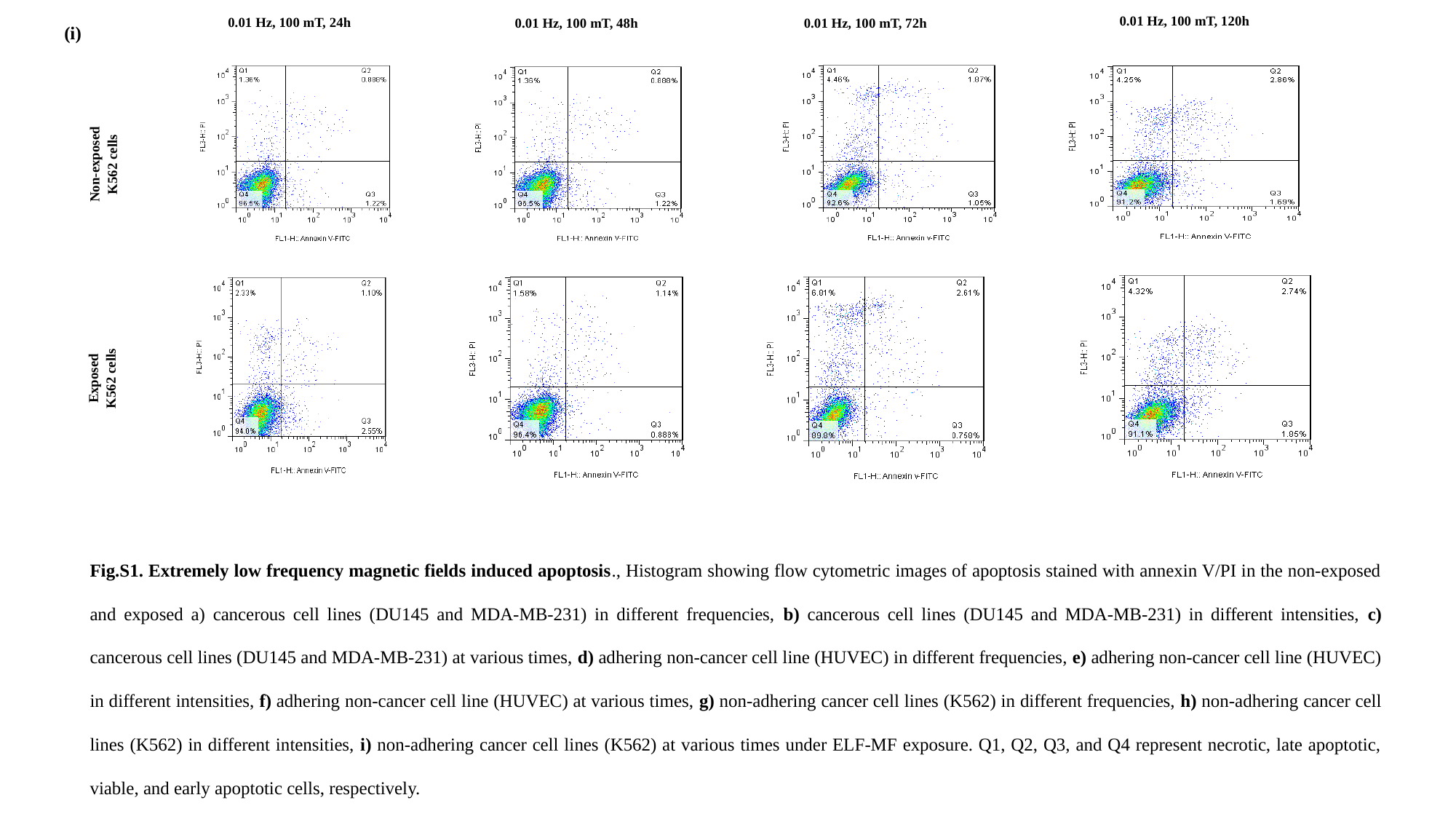

0.01 Hz, 100 mT, 120h
0.01 Hz, 100 mT, 24h
0.01 Hz, 100 mT, 48h
0.01 Hz, 100 mT, 72h
(i)
| | | | |
| --- | --- | --- | --- |
| | | | |
Non-exposed
K562 cells
Exposed
K562 cells
Fig.S1. Extremely low frequency magnetic fields induced apoptosis., Histogram showing flow cytometric images of apoptosis stained with annexin V/PI in the non-exposed and exposed a) cancerous cell lines (DU145 and MDA-MB-231) in different frequencies, b) cancerous cell lines (DU145 and MDA-MB-231) in different intensities, c) cancerous cell lines (DU145 and MDA-MB-231) at various times, d) adhering non-cancer cell line (HUVEC) in different frequencies, e) adhering non-cancer cell line (HUVEC) in different intensities, f) adhering non-cancer cell line (HUVEC) at various times, g) non-adhering cancer cell lines (K562) in different frequencies, h) non-adhering cancer cell lines (K562) in different intensities, i) non-adhering cancer cell lines (K562) at various times under ELF-MF exposure. Q1, Q2, Q3, and Q4 represent necrotic, late apoptotic, viable, and early apoptotic cells, respectively.

## Slide 12
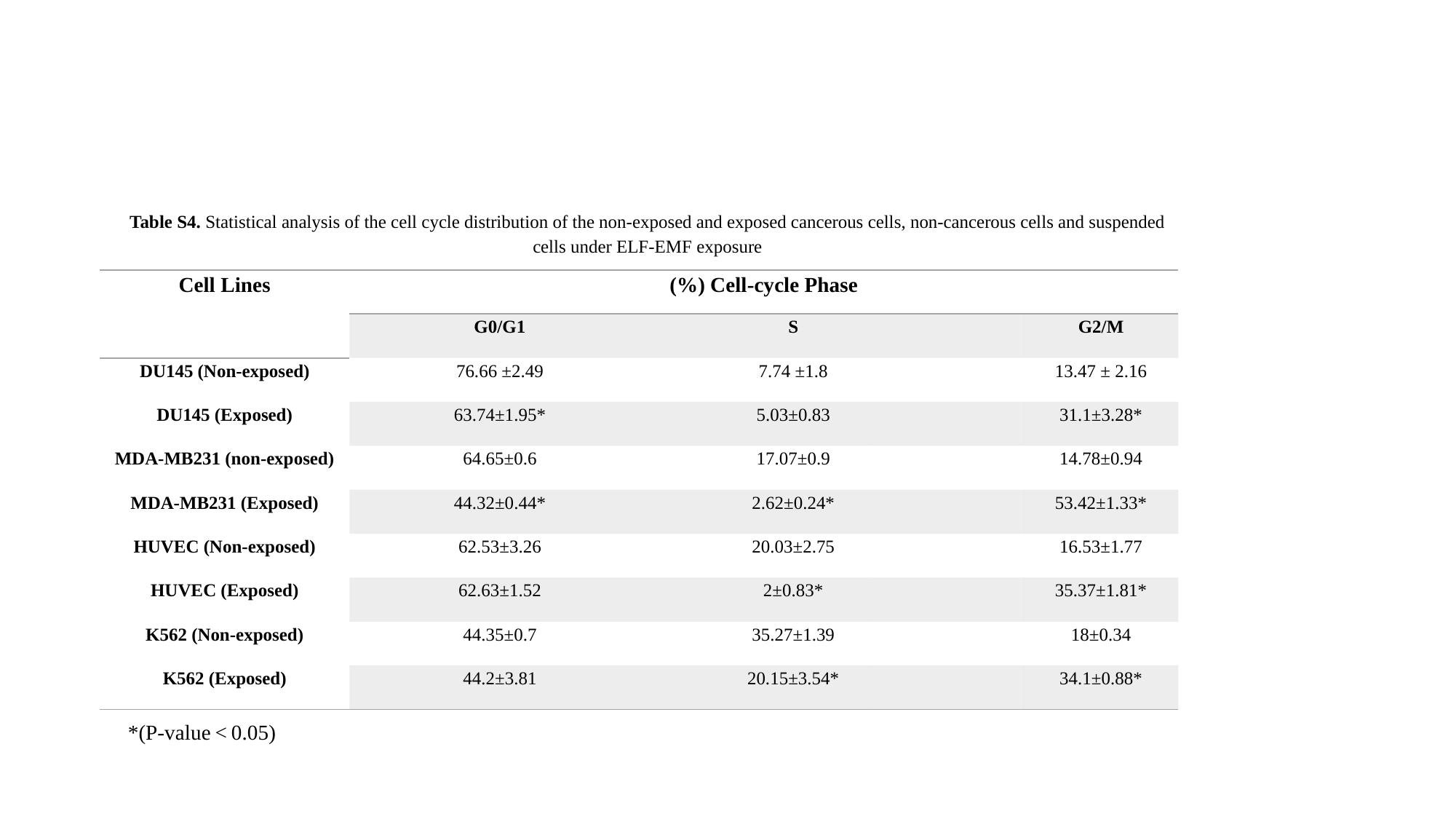

Table S4. Statistical analysis of the cell cycle distribution of the non-exposed and exposed cancerous cells, non-cancerous cells and suspended cells under ELF-EMF exposure
| Cell Lines | (%) Cell-cycle Phase | | | | | |
| --- | --- | --- | --- | --- | --- | --- |
| | | G0/G1 | | S | | G2/M |
| DU145 (Non-exposed) | | 76.66 ±2.49 | | 7.74 ±1.8 | | 13.47 ± 2.16 |
| DU145 (Exposed) | | 63.74±1.95\* | | 5.03±0.83 | | 31.1±3.28\* |
| MDA-MB231 (non-exposed) | | 64.65±0.6 | | 17.07±0.9 | | 14.78±0.94 |
| MDA-MB231 (Exposed) | | 44.32±0.44\* | | 2.62±0.24\* | | 53.42±1.33\* |
| HUVEC (Non-exposed) | | 62.53±3.26 | | 20.03±2.75 | | 16.53±1.77 |
| HUVEC (Exposed) | | 62.63±1.52 | | 2±0.83\* | | 35.37±1.81\* |
| K562 (Non-exposed) | | 44.35±0.7 | | 35.27±1.39 | | 18±0.34 |
| K562 (Exposed) | | 44.2±3.81 | | 20.15±3.54\* | | 34.1±0.88\* |
*(P-value < 0.05)

## Slide 13
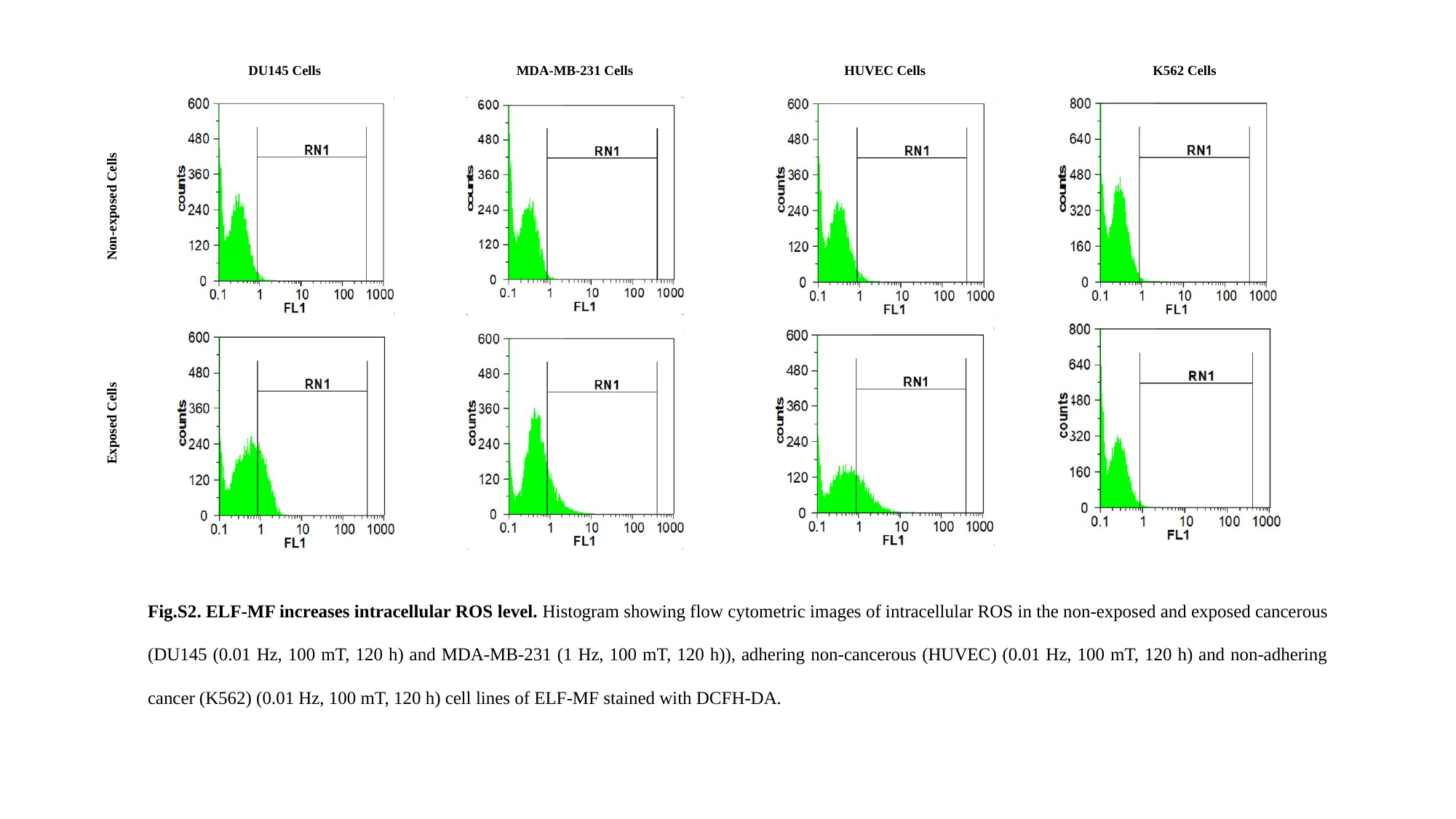

DU145 Cells
MDA-MB-231 Cells
HUVEC Cells
K562 Cells
| | | | |
| --- | --- | --- | --- |
| | | | |
Non-exposed Cells
Exposed Cells
Fig.S2. ELF-MF increases intracellular ROS level. Histogram showing flow cytometric images of intracellular ROS in the non-exposed and exposed cancerous (DU145 (0.01 Hz, 100 mT, 120 h) and MDA-MB-231 (1 Hz, 100 mT, 120 h)), adhering non-cancerous (HUVEC) (0.01 Hz, 100 mT, 120 h) and non-adhering cancer (K562) (0.01 Hz, 100 mT, 120 h) cell lines of ELF-MF stained with DCFH-DA.

## Slide 14
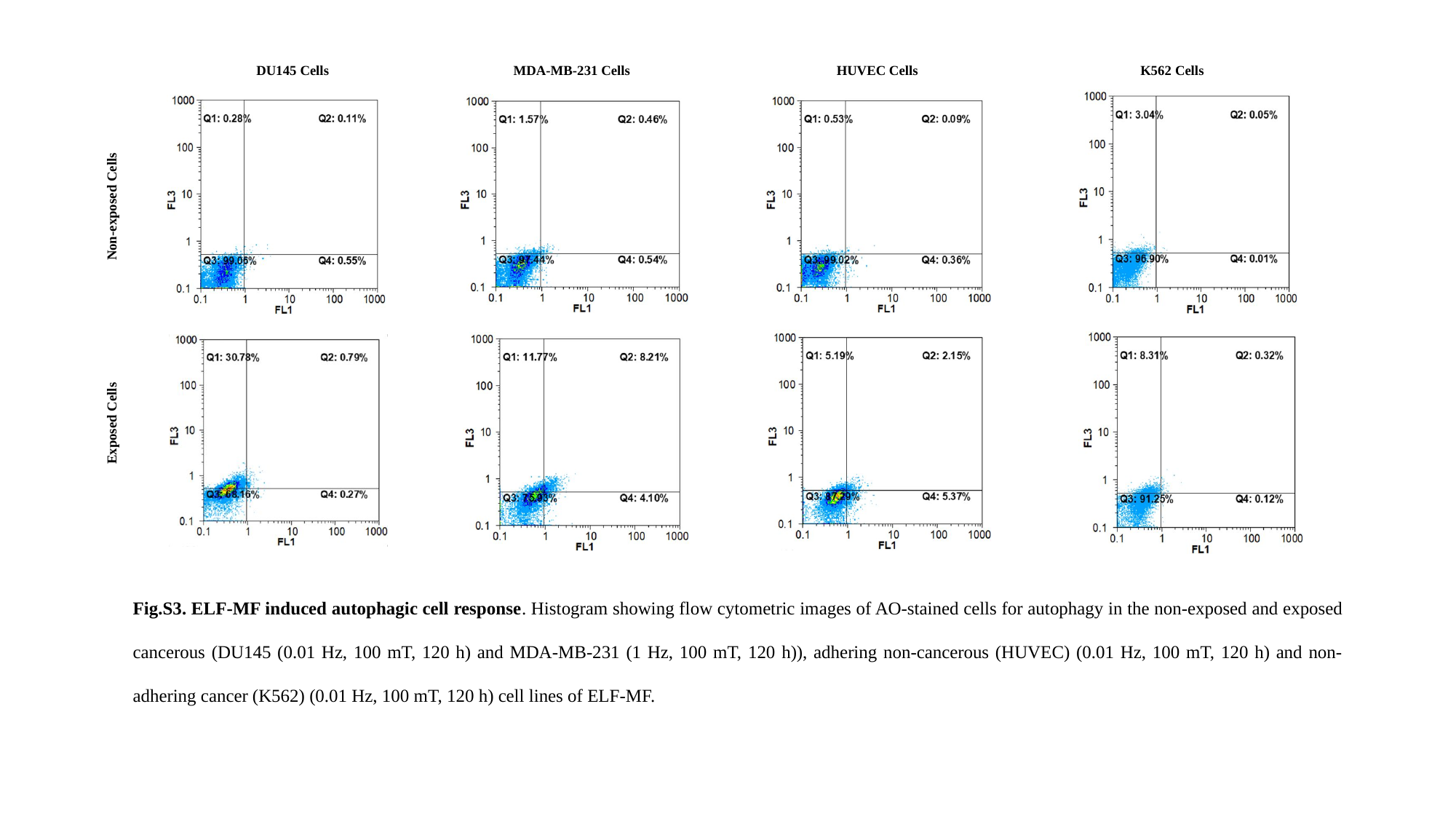

DU145 Cells
MDA-MB-231 Cells
HUVEC Cells
K562 Cells
| | | | |
| --- | --- | --- | --- |
| | | | |
Non-exposed Cells
Exposed Cells
Fig.S3. ELF-MF induced autophagic cell response. Histogram showing flow cytometric images of AO-stained cells for autophagy in the non-exposed and exposed cancerous (DU145 (0.01 Hz, 100 mT, 120 h) and MDA-MB-231 (1 Hz, 100 mT, 120 h)), adhering non-cancerous (HUVEC) (0.01 Hz, 100 mT, 120 h) and non-adhering cancer (K562) (0.01 Hz, 100 mT, 120 h) cell lines of ELF-MF.
